# *Staphylococcus aureus* exacerbates dermal IL-33/ILC2 axis activation through evoking RIPK3/MLKL-mediated necroptosis of dry skin

**DOI:** 10.1172/jci.insight.166821

**Published:** 2024-02-06

**Authors:** Chia-Hui Luo, Alan Chuan-Ying Lai, Chun-Chou Tsai, Wei-Yu Chen, Yu-Shan Chang, Ethan Ja-Chen Chung, Ya-Jen Chang

**Affiliations:** 1 Institute of Biomedical Sciences, Academia Sinica, Taipei, Taiwan.; 2 Taiwan International Graduate Program in Molecular Medicine, National Yang Ming Chiao Tung University and Academia Sinica, Taipei, Taiwan.; 3 Department of Biochemistry and Molecular Biology, National Cheng Kung University, Tainan, Taiwan.; 4 Institute of Microbiology and Immunology, National Defense University, Taipei, Taiwan.; 5 Institute of Translational Medicine and New Drug Development, China Medical University, Taichung, Taiwan.; 6 Graduate Institute of Medicine, College of Medicine, Kaohsiung Medical University, Kaohsiung, Taiwan.

**Keywords:** Immunology, Inflammation, Bacterial infections, Skin, Th2 response

## Abstract

Atopic dermatitis (AD) is a persistent skin disease typified by symptoms of dry skin and recurrent eczema. Patients with AD are at heightened risk for *Staphylococcus aureus* infection. Group 2 innate lymphoid cells (ILC2s) are mainly activated by epithelial cell–derived cytokines IL-33 and involved in the pathogenesis of AD. However, little is known about the effect of skin delipidization on the epithelial cell–derived cytokines and dermal ILC2s in AD. In our study, we investigated the mechanism by which *S*. *aureus* infection modulates and exacerbates the pathogenesis of dry skin, leading to type 2 inflammation in the context of innate immunity. In vivo, we found that *S*. *aureus* infection aggravated delipidization-induced dermal IL-33 release and dermal ILC2 accumulation, which exacerbated skin inflammation. We also noticed that *Il33^fl/fl^ K14^cre^* mice and *Tlr2^–/–^* mice exhibited attenuated skin inflammation. In vitro, treatment with necroptosis inhibitors reduced IL-33 release from *S*. *aureus*–infected keratinocytes. Mechanistically, we observed an increase in the necroptosis-associated kinases, MLKL and RIPK3, in *S*. *aureus*–infected mice, indicating that IL-33 release was associated with necroptotic cell death responses. Our results reveal that *S*. *aureus* infection–elicited keratinocyte necroptosis contributes to IL-33–mediated type 2 inflammation, which exacerbates the pathogenesis of dry skin.

## Abstract 2

*S. aureus* exacerbates skin inflammation by augmenting IL-33 release from keratinocytes, which is dependent on TLR2 signaling and necroptosis.

## Graphical abstract

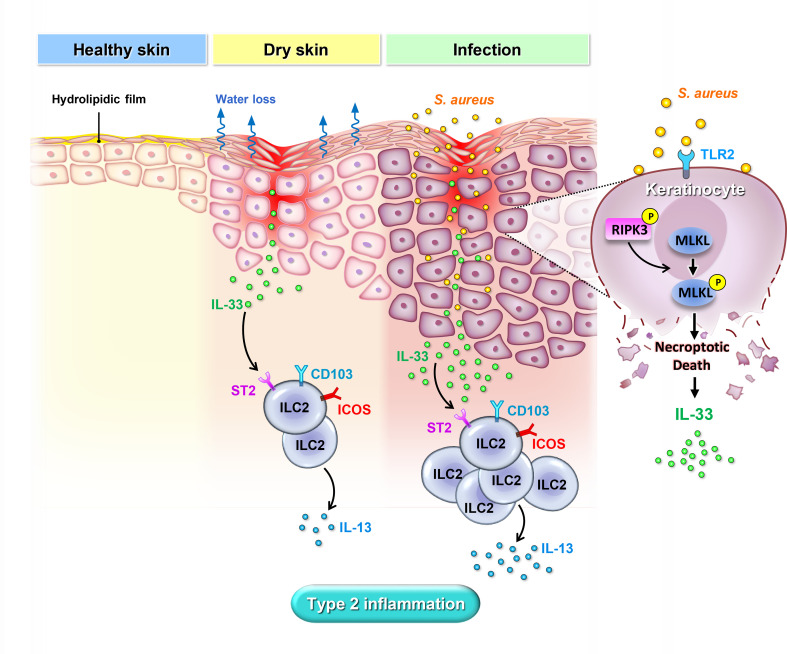

## Introduction

Atopic dermatitis (AD) is a common chronic skin disorder characterized by dry skin, itching, and recurrent eczematous skin lesions, and a dominant Th cell (Th2) response is observed in the acute phase of AD (1, 2). Skin lesions in patients with acute AD exhibit epidermal thickening and dermal leukocyte infiltration (3), and the degree of skin barrier disruption in patients with AD correlates with disease severity (4). Mechanical skin injury, caused by the removal of lipid components and scratching, aggravates the defect in skin barrier function, leading to the development of dry skin and the release of cytokines that drive the immune response (5–7). Recent studies have also reported that the skin microbiome plays a role in the pathogenesis of AD (8, 9). Notably, *Staphylococcus aureus* often colonizes and infects the skin of patients with AD, showing a strong association with disease severity (10, 11). Importantly, defects in the epidermal lipid barrier increase susceptibility to *S*. *aureus* infection in skin lesions and correlate with the overexpression of Th2 cytokines in patients with AD (11, 12).

Group 2 innate lymphoid cells (ILC2s) are Lin^–^CD45^+^Th1.2^+^CD127^+^ICOS^+^ cells that express ST2, the receptor for the IL-33, and their development and function are regulated by the transcription factor GATA3 (13). ILC2s are tissue-resident innate-like lymphocytes that contribute to the initiation and maintenance of Th2 cytokine–driven immune responses (14). Epithelial cell–derived cytokines, IL-33 and thymic stromal lymphopoietin (TSLP), are important activators of ILC2s. They elicit a rapid and robust type 2 inflammatory immune response, driven by IL-13 and IL-5 production, in both mouse and human ILC2s (15). Specifically, ILC2-derived IL-13 and IL-5 are necessary and sufficient for the development of AD-like symptoms in a mouse model (16, 17). The levels of ILC2s and type 2 cytokines are also significantly elevated in skin lesions of patients with AD (18).

Necroptosis is a regulated form of necrotic death that involves the formation of a necrosome complex composed of receptor-interacting protein 1 and 3 (RIP1/RIP3) and the mixed-lineage kinase domain-like pseudokinase (MLKL) (19). The RIP1/RIP3 complex recruits and phosphorylates MLKL, forming the necrosome. This phosphorylation of MLKL causes a conformational change, leading to its oligomerization and subsequent translocation to the plasma membrane, forming large pores that permit ion influx, potentially causing cell swelling and membrane lysis (19). The necroptosis pathway can be triggered by various signals, including the ligation of death receptors like Fas, TNFR, IFNs, TLRs, and intracellular RNA- or DNA-sensing molecules (19). While the stimulation of death receptors can instigate different forms of cell death, depending on the assembly of regulatory proteins, it remains uncertain whether necroptosis plays a role in *S*. *aureus*–induced pathogenesis of skin inflammation. Additionally, the link between necroptosis, ILC2s, and associated type 2 cytokines in the overall pathogenesis of AD requires further clarification. Therefore, this study aims to investigate the mechanism by which *S*. *aureus* infection modulates and exacerbates the pathogenesis of dry skin toward type 2 inflammation in the context of innate immunity.

## Results

### Delipidization induces type 2 skin inflammation in young mice.

**Figure 1:**
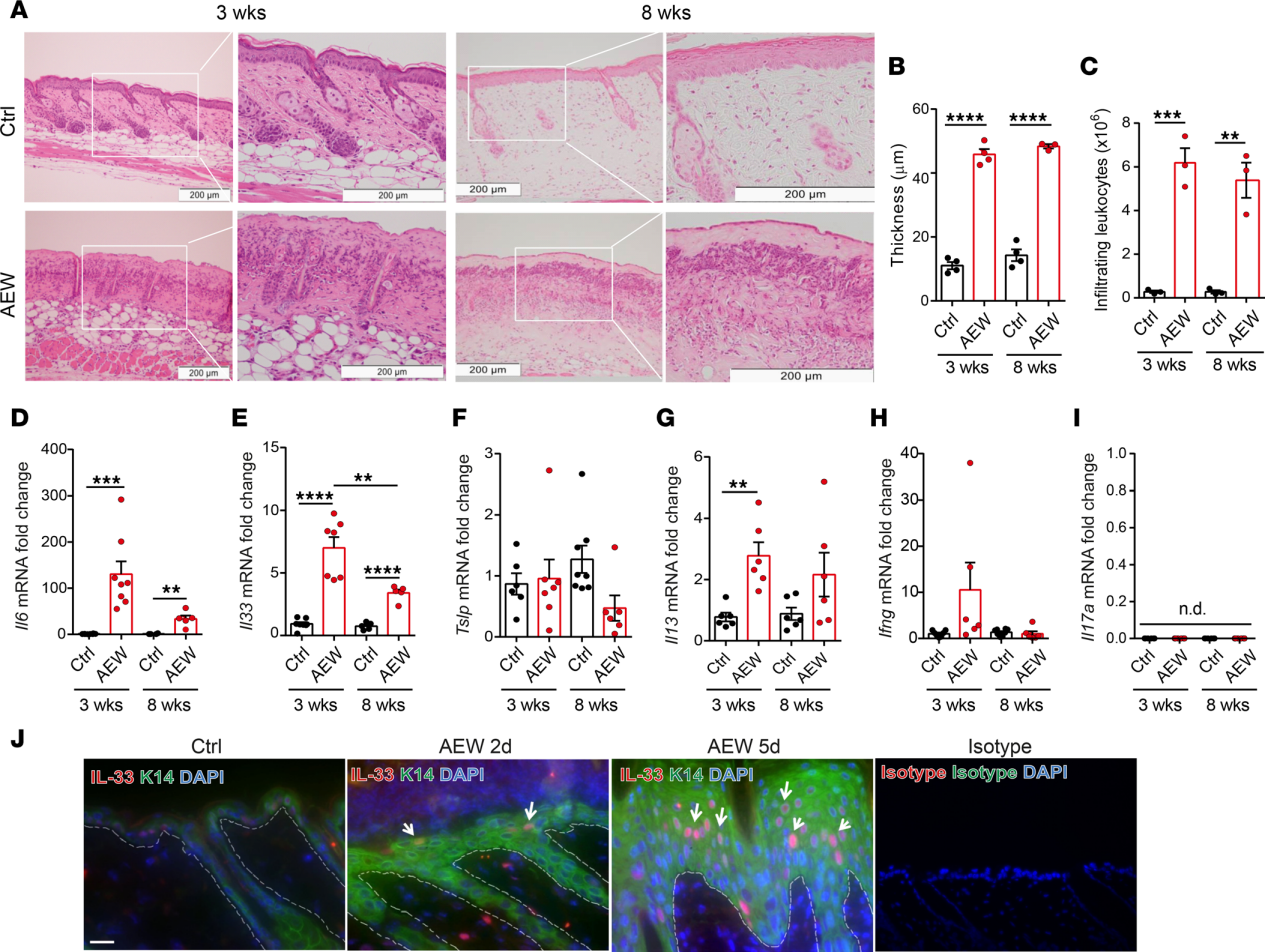
Delipidization induces skin inflammation and epithelium-derived cytokine expression. C57BL/6 mice were subjected to AEW treatment twice daily for 2 days and were sacrificed 1 day after the last treatment. (**A**) H&E-stained skin section. Scale bars: 200 μm. Images to the right are magnified views of regions boxed in images on the left. (**B**) Measurement of skin thickness. (**C**) Total numbers of CD45^+^ leukocytes in skin. (**D**–**I**) mRNA levels of epithelial cell–derived cytokines *Il6* (**D**), *Il33* (**E**), *Tslp* (**F**), *Il13* (**G**), *Ifng* (**H**), and *Il17a* (**I**) in delipidized skin. (**J**) Image of immunofluorescence staining in mice skin. Scale bars: 50 μm. Dotted lines indicate the border between epidermis and dermis. Arrows indicate keratinocytes with IL-33 expression. Data are shown as mean ± SEM from 3 independent experiments (*n* = 3-8 per group). Statistical analysis was performed using one-way ANOVA (**B**–**I**). *n.d*. Not detectable. **P < 0.01, ***P < 0.001, ****P < 0.0001.

Previous studies have reported that treatment of mouse skin with a mixture of acetone and ether followed by water (AEW) causes skin delipidization and triggers spontaneous scratching in mice (5, 6). To determine the effects of delipidization, we performed AEW treatment on the skin of 3-week-old (young) and 8-week-old (adult) WT mice (Supplemental Figure 1A; supplemental material available online with this article; https://doi.org/10.1172/jci.insight.166821DS1). We discovered that delipidization, achieved through AEW treatment, induced skin barrier disruption. This was evidenced by increased transepidermal water loss (TEWL), epidermal thickening, skin leukocyte infiltration, and elevated *Il6* mRNA expression in both young and adult mice (Supplemental Figure 1B and Figure 1, A–D). Additionally, using Oil Red O staining, we noted a reduction in lipid components on the epidermis surface following AEW treatment (Supplemental Figure 1C). Skin barrier proteins, such as Filaggrin, Claudin 1, and Involucrin, were also observed to be downregulated after AEW treatment (Supplemental Figure 1, D–F), suggesting that delipidization compromises skin integrity.

Next, we investigated whether delipidization affects cytokines production in the dorsal skin of mice. The mRNA levels of *Il33* and *Il13*, which encode Th2 cytokines, increased in the delipidized skin, whereas the expression of *Tslp*, *Ifng* (which encodes a Th1 cytokine), and *Il17a* (which encodes a Th17 cytokine) were unaltered after AEW treatment (Figure 1, E–I). We further examined the protein expression of IL-33 using immunofluorescence staining after the 2- or 5-day AEW treatment and observed a substantial increase over time (Figure 1J). We also noticed that the epidermal thickening of 5-day AEW treatment was more severe than 2-day AEW treatment (Supplemental Figure 1, G and H). In addition, levels of *Il33*, *Il5*, and *Il13* mRNAs increased 2 days after treatment and subsided on day 5, whereas the expression of *Tslp* mRNA remained unaffected throughout the assay period (Supplemental Figure 1, I–L). Expression of other type 2 cytokines, such as IL-4 and IL-5, also increased in the delipidized skin. However, the expression of IL-9 was not affected by delipidization (Supplemental Figure 2, A–C).

Tape stripping is a simple and efficient method for disrupting the skin barrier (20). Therefore, we compared the changes in the expression of *Il33*, *Il13*, *Ifng*, and *Il17a* between the tape stripping and the delipidization treatments. We found that the delipidization treatment induced higher *Il33* and *Ifng* mRNA expressions than tape stripping, whereas the mRNA expressions of *Il13* and *Il17a* did not differ significantly between groups (Supplemental Figure 2, D–G). These data suggest that delipidization induces skin barrier disruption and skin inflammation, characterized by leukocyte infiltration, epidermal thickening, and increased levels of *Il33* and *Il13* in the skin of mice.

### Keratinocyte-derived IL-33 contributes to skin inflammation.

**Figure 2:**
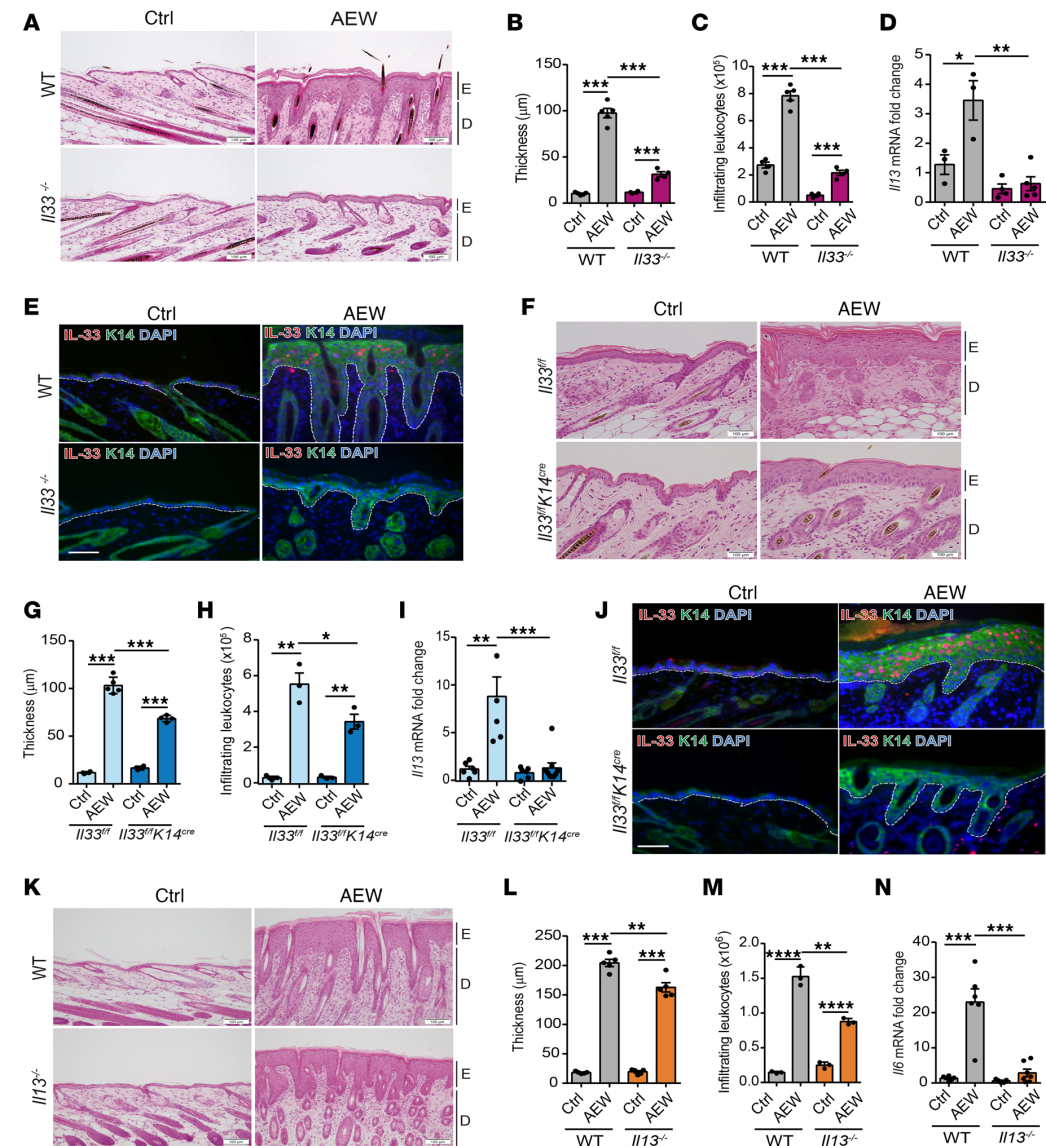
Keratinocyte-derived IL-33 participates in skin inflammation and regulates IL-13 and IL-6 expression. Three-week-old mice were treated with AEW twice daily for 2 days and were sacrificed 1 day after the last treatment; controls were treated with water. (**A**) H&E-stained skin sections. (**B**) Measurement of skin thickness. (**C**) Total numbers of CD45^+^ leukocytes in skin. (**D**) mRNA of *Il13* in skin. (**E**) Image of immunofluorescence staining in skin. (**F**) H&E-stained skin sections. (**G**) Measurement of skin thickness. (**H**) Total numbers of CD45^+^ leukocytes in skin. (**I**) mRNA of *Il13* in skin. (**J**) Image of immunofluorescence staining in skin. (**K**) H&E-stained skin sections. (**L**) Measurement of skin thickness. (**M**) Total numbers of CD45^+^ leukocytes in skin. (**N**) mRNA of *Il6* in skin. Data are shown as mean ± SEM from 3 independent experiments (*n* = 3–6 per group). Statistical analysis was performed using 1-way ANOVA (**B**–**D**, **G**–**I**, and **L**–**N**). *P < 0.05, **P < 0.01, ***P < 0.001, ****P < 0.0001. Scale bars: 100 μm. E, epidermidis; D, dermal. Dotted lines indicate the border between the epidermis and dermis

To further study the role of IL-33 in skin inflammation, we generated IL-33–KO (*Il33^–/–^*) mice and keratinocyte-specific IL-33–KO mice (*Il33^fl/fl^ K14^cre^*). The specificity of IL-33 deletion in keratinocytes was confirmed using immunofluorescence staining (Supplemental Figure 3). Global deletion of IL-33 reduced skin thickness, the number of infiltrating leukocytes, and *Il13* expression in the skin of *Il33^–/–^* mice compared with WT mice (Figure 2, A–E). Moreover, the TEWL level was also found to be decreased in *Il33^–/–^* mice (Supplemental Figure 4A). Similar results were observed in mice with a keratinocyte-specific KO of IL-33 (*Il33^fl/fl^ K14^cre^*), including downregulation of epidermal thickening, infiltrating leukocytes, *Il13* expression, and TEWL in the skin of *Il33^fl/fl^ K14^cre^* mice (Figure 2, F–J, and Supplemental Figure 4B).

Next, to evaluate how IL-13 affects skin inflammation, we examined the effects of delipidization in *Il13^–/–^* mice. We observed that epidermal thickening, leukocyte infiltration, and TEWL were less pronounced in *Il13^–/–^* mice (Figure 2, K–M, and Supplemental Figure 4C). Moreover, the level of mRNA encoding the proinflammatory cytokine IL-6 was also reduced in *Il13^–/–^* mice compared with WT mice (Figure 2N). These results suggest that keratinocyte-derived IL-33 regulates IL-13 expression, leading to the manipulation of skin inflammation induced by delipidization.

### Delipidization induces dermal ILC2 accumulation independent of adaptive immunity.

**Figure 3:**
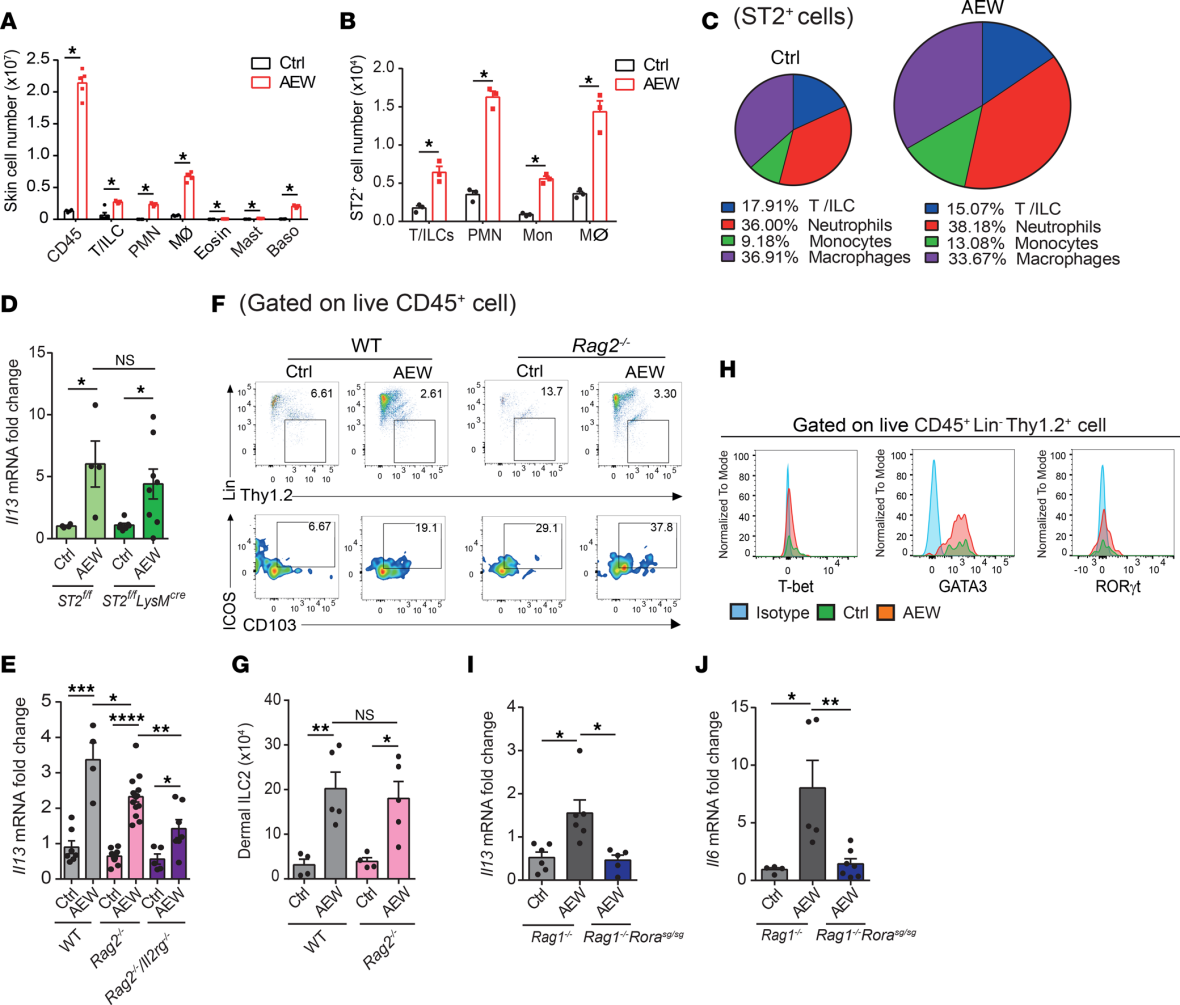
Delipidization induces dermal ILC2 accumulation. (**A**–**C**) Three-week-old C57BL/6 mice were treated with AEW for 2 days; controls were treated with water. (**A**) Total number of CD45^+^ cells, T cells, and innate lymphoid cells (T/ILC), neutrophils (PMN), macrophages (Mϕ), eosinophils (Eosin), mast cells (Mast), and basophils (Baso) in the skin. (**B**) Total number of ST2^+^ cells of indicated types in the skin. (**C**) Pie charts depicting the relative proportions of lymphocytes and myeloid cells within the total ST2^+^ cells present in skin. (**D** and **E**) Levels of *Il13* in the skin of 3-week-old *ST2^fl/fl^* and *ST2^fl/fl^LysM^cre^* mice (**D**) and *Rag2^–/–^* and *Rag2^–/–^ Il2rg^–/–^* mice (**E**) treated or not with AEW for 2 days. (**F** and **G**) Three-week-old WT and *Rag2^–/–^* mice were treated with AEW for 2 days; controls were treated with water. Representative FACS analysis and quantitation of cell numbers of dermal ILC2s (CD45^+^Lin^–^Thy1.2^+^GATA3^+^ICOS^+^CD103^+^) in skin. (**H**) Representative FACS analysis of T-bet, GATA3, and RORγt expression in dermal ILCs (CD45^+^Lin^–^Thy1.2^+^ICOS^+^CD103^+^) in the skin of WT mice. (**I** and **J**) Three-week-old *Rag1^–/–^* or *Rag1^–/–^ Rora^sg/sg^* mice were treated with AEW for 2 days; controls were treated with water. Levels *Il13* (**I**) and *Il6* (**J**) mRNA quantified in the skin. Data are shown as mean ± SEM from 3 independent experiments (*n* = 3–12 per group). Statistical analysis was performed using 1-way ANOVA (**A**, **B**, **D**, **E**, **G**, **I**, and **J**). *P < 0.05, **P < 0.01, ***P < 0.001, ****P < 0.0001.

To evaluate whether dermal ILC2s influence skin inflammation, we performed an analysis of skin-infiltrating leukocytes after delipidization. We found that the total CD45^+^ leukocyte numbers increased considerably after AEW treatment (Figure 3A). By gating on various lymphocyte (T cells and ILCs) and myeloid cell (neutrophils, macrophages, eosinophils, mast cells, and basophils) subsets (Supplemental Figure 5A), we found that macrophages, neutrophils, T cells, ILCs, and basophils were the major cell populations that were induced upon treatment with AEW (Figure 3A and Supplemental Figure 5B). Furthermore, the expression of the IL-33 receptor ST2 were higher in lymphocytes, neutrophils, monocytes, and macrophages in the skin of delipidized mice compared with those in the control group (Figure 3, B and C, and Supplemental Figure 5C). To exclude the effect of IL-33 signaling in myeloid cells, we generated *ST2^fl/fl^LysM^cre^* mice, which lack ST2 in macrophages and neutrophils. We found that ST2 deficiency in myeloid cells neither affected *Il13* expression (Figure 3D) nor altered TEWL levels in delipidized skin (Supplemental Figure 6A).

To further clarify the role of T cells and ILCs in delipidization-induced skin inflammation, we compared the effect of delipidization in WT, *Rag2*^–/–^ mice (which lack T and B cells), and *Rag2^–/–^ Il2rg^–/–^* mice (which lack T cells, B cells, and ILCs) and found that the expression of *Il13* and the TEWL levels in the skin of *Rag2^–/–^ Il2rg^–/–^* mice were lower than that in WT and *Rag2*^–/–^ mice (Figure 3E and Supplemental Figure 6B). These results demonstrate that T cells and ILCs are likely to participate in skin inflammation caused by delipidization with no or minimal input from the myeloid cells.

In 2013, Roediger et al. reported that dermal ILC2s represent a unique population expressing the CD103 surface marker (21). In line with this report, we confirmed the expression of CD103 in ILC2s from the inflamed skins of WT and *Rag2^–/–^* mice (Figure 3, F and G). Among the innate lymphoid subsets, we further identified ILC2s as the predominant dermal population (CD45^+^Lin^–^Thy1.2^+^ICOS^+^CD103^+^) based on GATA3 expression (Figure 3H). A previous study suggested that deletion of the transcription factor RORα results in the failure of ILC2s to expand in response to IL-33 (22). To evaluate whether this is the case in our system, we treated *Rag1^–/–^ Ror*α*^sg/sg^* mice with AEW and analyzed the expression of *Il13* in samples of the inflamed skin. The levels of expression of *Il13* and *Il6* mRNAs were lower in *Rag1^–/–^ Ror*α*^sg/sg^* mice than *Rag1^–/–^* mice (Figure 3, I and J). Furthermore, the TEWL levels were also decreased in the *Rag1^–/–^ Ror*α*^sg/sg^* mice (Supplemental Figure 6C). Taken together, these results suggest that delipidization induces the accumulation of dermal ILC2s, contributing to skin inflammation through IL-13 production.

### Keratinocyte-derived IL-33 drives IL-13^+^ dermal ILC2 activation.

**Figure 4:**
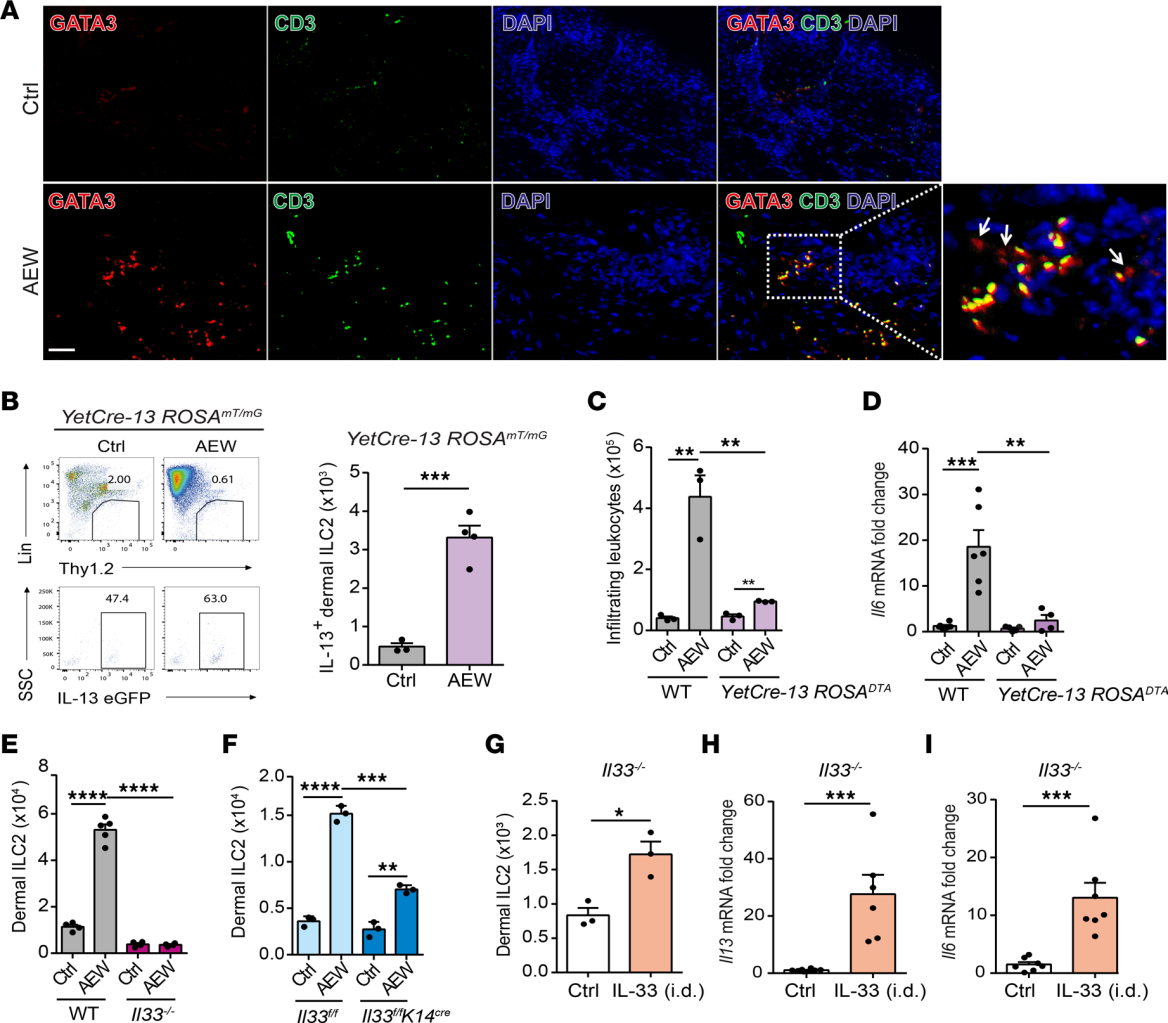
Keratinocyte-derived IL-33 drives IL-13^+^ dermal ILC2 activation. (**A**–**F**) Three-week-old mice were treated with AEW twice daily for 2 days and sacrificed 1 day after the last treatment; controls were treated with water. (**A**) Immunofluorescence staining of CD3 (green), GATA3 (red), and DAPI (blue) in skin lesions of WT mice. Scale bars: 100 μm. Arrows indicate dermal ILC2s (CD3^–^GATA3^+^). (**B**) Representative FACS analysis and quantitation of numbers of IL-13–producing dermal ILC2s in the skin of *YetCre-13 ROSA*^mT/mG^ mice. (**C**) Mean numbers of total CD45^+^ leukocytes in the skin of *YetCre-13 ROSA^DTA^* mice. (**D**) Levels of *Il6* mRNA in the skin of *YetCre-13 ROSA^DTA^* mice. (**E** and **F**) Numbers of dermal ILC2s in WT and *Il33^–/–^* mice (**E**) and *Il33^fl/fl^* and *Il33^fl/fl^ K14^cre^* mice (**F**). (**G**–**I**) Three-week-old *Il33^–/–^* mice were administered 1 μg of IL-33 recombinant proteins intradermally once daily for 3 days and were sacrificed 1 day after the last treatment; controls received vehicle. (**G**) Number of dermal ILC2s in control and treated mice. (**H**) Levels of *Il13* mRNA in the skin. (**I**) Levels of *Il6* mRNA in the skin. Data are shown as mean ± SEM from 3 independent experiments (*n* = 3–7 per group). Statistical analysis was performed using 1-way ANOVA (**C**–**F**) or an unpaired 2-tailed *t* test (**B** and **G**–**I**). *P < 0.05, **P < 0.01, ***P < 0.001, ****P < 0.0001.

To verify the infiltration of dermal ILC2s into the delipidized skin, we used fluorescence microscopy and found that dermal ILC2s, identified as GATA3^+^CD3^–^, accumulated in the dermis region of the skin (Figure 4A). To discern the role of inflammatory dermal ILC2s, the *YetCre-13 ROSA^mT/mG^* dual fluorescent protein reporter mouse, in which IL-13–expressing cells are labeled with a chromatin-specific enhanced green fluorescence protein upon activation by Cre-mediated DNA recombination, was utilized to trace IL-13–producing cells. We observed that IL-13^+^ dermal ILC2s (eGFP^+^) accumulated in the dermis of the *YetCre-13 ROSA^mT/mG^* reporter mice after delipidization (Figure 4B). To confirm the importance of these IL-13–producing cells, we subjected *YetCre-13 ROSA^DTA^* mice, which are depleted of IL-13–producing cells upon induction, to delipidization. Deficiency in IL-13–producing cells resulted in decreased infiltrating leukocyte accumulation and reduced IL-6 expression (Figure 4, C and D). Moreover, the accumulation of dermal ILC2s were lower in *Il33*^–/–^ and *Il33^fl/fl^ K14^cre^* mice than in their respective WT littermates (Figure 4, E and F). To further ascertain the role of keratinocyte-derived IL-33 in skin inflammation, we administered recombinant IL-33 protein to *Il33*^–/–^ mice via the intradermal route. We found that exogenous IL-33 injection did not affect epidermal thickness but increased the level of TEWL in *Il33*^–/–^ mice (Supplemental Figure 7, A–C). Moreover, the dermal ILC2 accumulation and the levels of *Il13* and *Il6* mRNAs were elevated in the skin of *Il33*^–/–^ mice treated with IL-33 injection compared with untreated mice (Figure 4, G–I). Taken together, our data suggest that IL-33 derived from keratinocytes induces dermal ILC2 activation and accumulation and elicits IL-6 expression.

### S. aureus enhances IL-33 expression.

**Figure 5:**
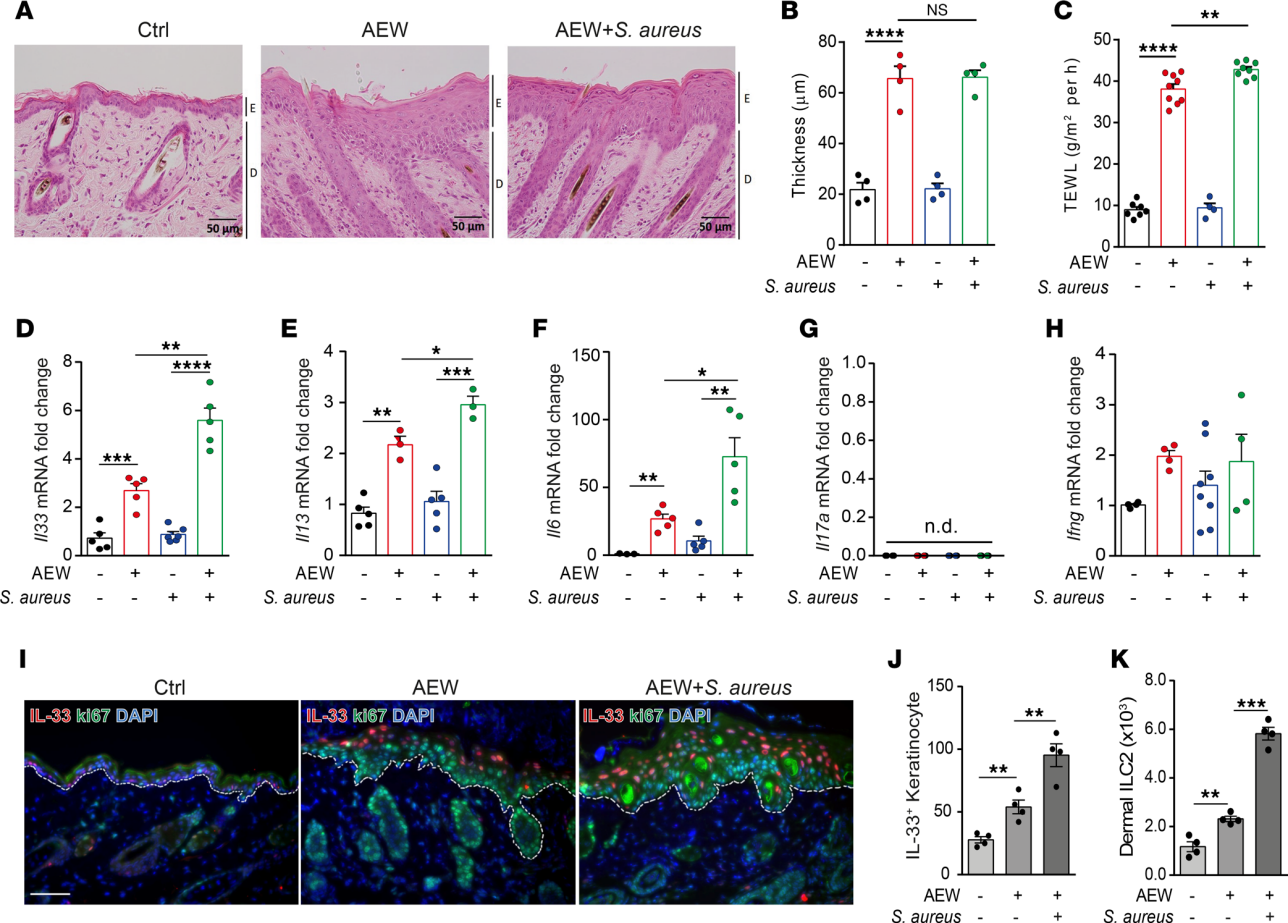
*S*. *aureus* infection exacerbates delipidization-induced IL-33 expression and dermal ILC2 infiltration. (**A**–**K**) WT mice were epicutaneously challenged with *S*. *aureus* with or without delipidization. (**A**) H&E-stained skin sections. Scale bars: 50 μm. (**B**) Measurement of skin thickness. (**C**) Transepidermal water loss (TEWL) in skin. (**D**–**H**) Levels of *Il33* (**D**), *Il13* (**E**), *Il6* (**F**), *Il17a* (**G**), and *Ifng* (**H**) mRNAs in the skin. (**I**) Immunofluorescence staining of IL-33 (red), Ki67 (green), and DAPI (blue) in the skin. Scale bars: 50 μm. (**J**) Number of IL-33^+^ keratinocytes in the skin. (**K**) Number of ILC2s (CD45^+^Lin^–^Thy1.2^+^GATA3^+^ICOS^+^CD103^+^) in the skin. Data are shown as mean ± SEM from 3 independent experiments (*n* = 3–10 per group). Statistical analysis was performed using 1-way ANOVA (**B**–**H**, **J**, and **K**). *P < 0.05, **P < 0.01, ***P < 0.001, ****P < 0.0001.

*S*. *aureus* reportedly colonizes skin lesions and aggravates disease severity in patients with AD (18). However, the effect of *S*. *aureus* isolated from patients with AD on keratinocytes is still unclear. To determine whether *S*. *aureus* contributes to the exacerbation of delipidization-induced skin inflammation, we epicutaneously challenged C57BL/6 mice with *S*. *aureus* under delipidization. We found that the epidermal thickness was not altered after *S*. *aureus* infection. However, the level of TEWL was partially increased in *S*. *aureus*–infected skin compared with AEW treatment alone (Figure 5, A–C). We next investigated the expression of epithelial cell–derived cytokines after *S*. *aureus* infection and found that *S*. *aureus* infection aggravated delipidization-induced *Il33*, *Il13*, and *Il6* mRNA levels in the skin (Figure 5, D–F). However, the level of *Il17a* mRNA was undetectable under all conditions, and the level of *Ifng* mRNA was not significantly changed versus the control under any condition (Figure 5, G and H). Furthermore, we observed that *S*. *aureus* infection augmented IL-33 expression and increased dermal ILC2 accumulation (Figure 5, I–K). Overall, these results suggest that delipidization in conjunction with *S*. *aureus* infection exacerbates IL-33 and IL-13 induction and dermal ILC2 infiltration.

### IL-33 deficiency in keratinocytes attenuates S. aureus–induced skin inflammation.

**Figure 6:**
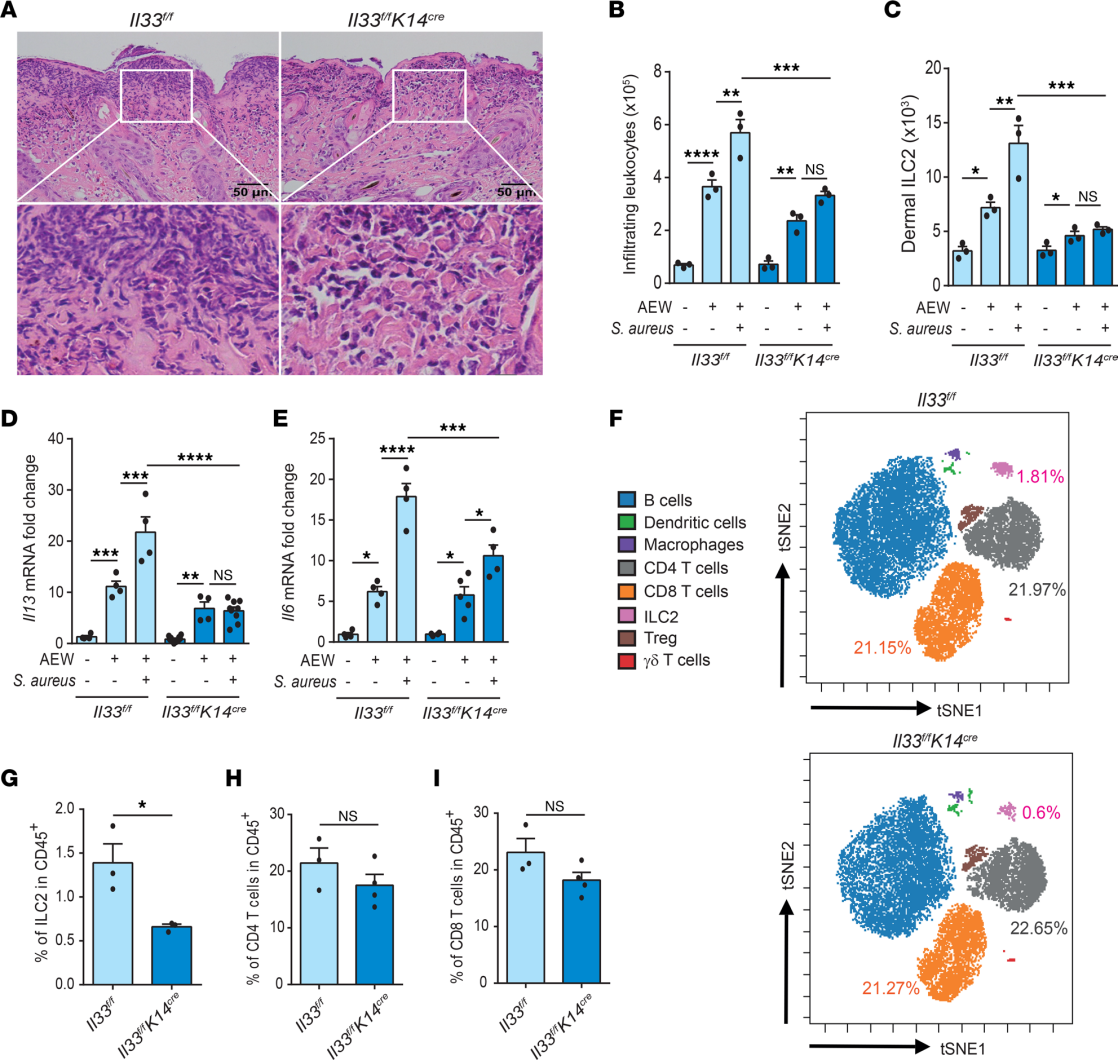
Keratinocyte-derived IL-33 regulates skin inflammation aggravated by *S*. *aureus*. (**A**–**I**) Three-week-old *Il33^fl/fl^* and *Il33^fl/fl^ K14^cre^* mice were epicutaneously challenged with *S*. *aureus* (5 × 10^8^ CFU) under delipidization treatment for 24 hours. (**A**) H&E-stained skin sections. Scale bars: 50 μm. (**B**) Mean numbers of total CD45^+^ leukocytes in the skin. (**C**) Number of dermal ILC2s (CD45^+^Lin^–^Thy1.2^+^GATA3^+^ICOS^+^CD103^+^) in the skin. (**D**) Levels of *Il13* mRNA in the skin. (**E**) Levels of *Il6* mRNA in the skin. (**F**) The distribution of CD45^+^ cell subsets in skin draining lymph node leukocytes determined by mass cytometry visualized in a tSNE map. The maps are color-coded by cell identities. (**G**–**I**) Frequencies of ILC2 (**G**), CD4^+^ T cells (**H**), and CD8^+^ T cells (**I**) in CD45^+^ cell subsets determined by mass cytometry. Data are shown as mean ± SEM from 3 independent experiments (*n* = 3–6 per group). Statistical analysis was performed using 1-way ANOVA (**B**–**E**) or an unpaired 2-tailed *t* test (**G**–**I**). *P < 0.05, **P < 0.01, ***P < 0.001, ****P < 0.0001.

To assess the importance of keratinocyte-derived IL-33, we epicutaneously challenged *Il33^fl/fl^* and *Il33^fl/fl^ K14^cre^* mice with *S*. *aureus* after delipidization. We observed that leukocyte infiltration and expression of *Il13* and *Il6* mRNAs were significantly lower in *Il33^fl/fl^ K14^cre^* mice compared with littermate controls (Figure 6, A, B, D, and E). In accord with this, fewer dermal ILC2s accumulated in the skin of these mice (Figure 6C). We then performed CyTOF analysis to examine the role of IL-33 in immune cell distribution in the skin draining lymph nodes. Under *S*. *aureus* infection, we observed a significant decrease in ILC2 frequency in *Il33^fl/fl^ K14^cre^* mice compared with their littermate controls. However, the frequencies of CD4 T cells and CD8 T cells were unaffected among the groups (Figure 6, F–I, and Supplemental Table 1). Collectively, our data suggest that IL-33 derived from keratinocytes drives skin inflammation during *S*. *aureus* infection in delipidized skin.

### The increase in IL-33 release from keratinocytes induced by S. aureus is dependent on TLR2 signaling.

**Figure 7:**
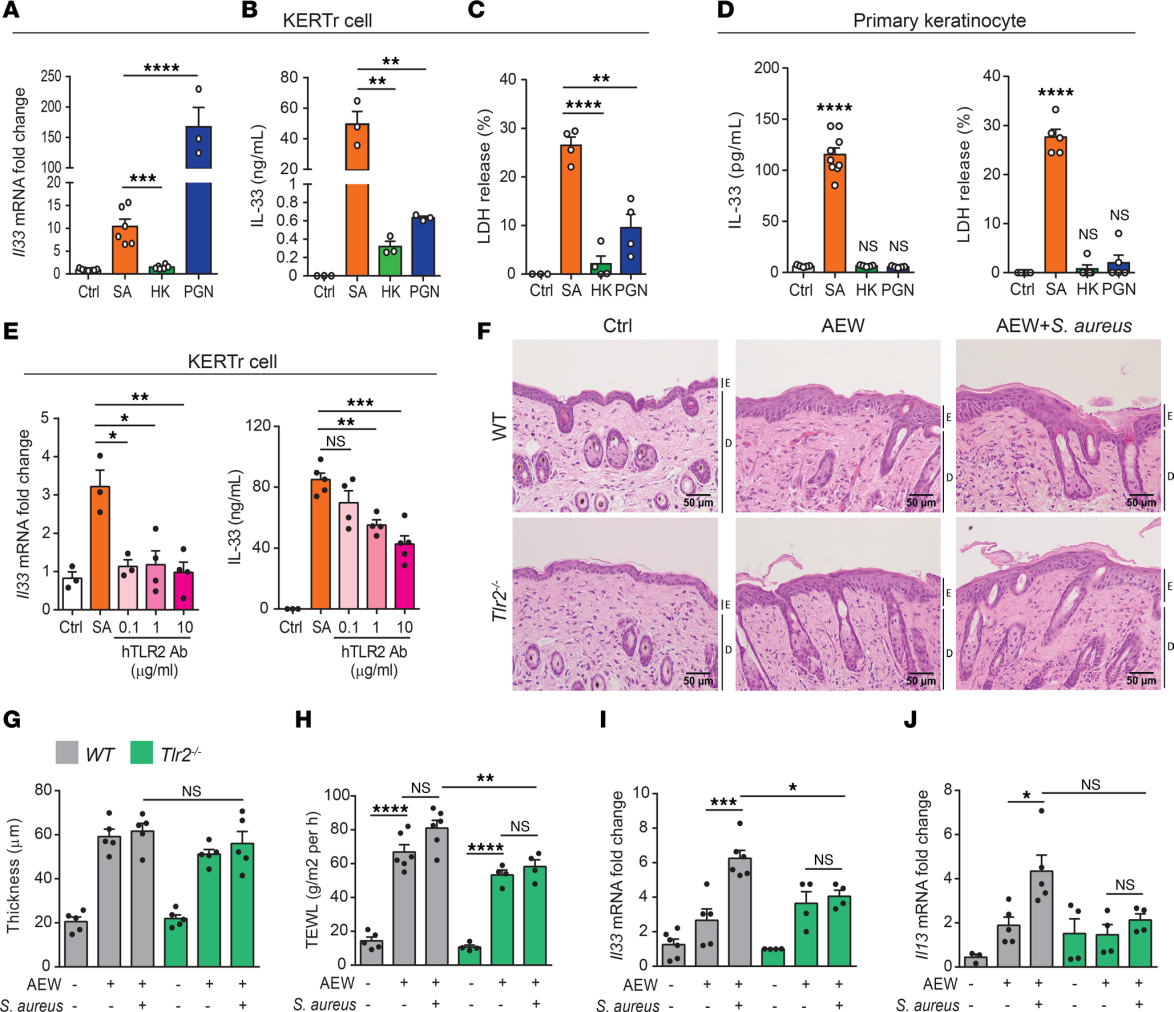
*S*. *aureus*–induced IL-33 release from keratinocytes is dependent on TLR2 signaling. (**A**–**D**) Levels of IL-33 and percentage of LDH released from KERTr cells (**A**–**C**) and mouse primary keratinocytes (**D**) infected with *S*. *aureus* (SA) or heat-killed *S*. *aureus* (HK), or treated with peptidoglycan from *S*. *aureus* (PGN). (**E**) mRNA level and protein level of IL-33 in KERTr cells infected with *S*. *aureus* and treated or not with TLR2 neutralizing antibody. (**F**–**J**) Three-week-old WT and *Tlr2^–/–^* mice were epicutaneously challenged with *S*. *aureus* (5 × 10^8^ CFU) under delipidization treatment for 24 hours. (**F**) H&E-stained skin sections. Scale bars: 50 μm. E, epidermidis; D, dermal. (**G**) Measurement of skin thickness. (**H**) Transepidermal water loss (TEWL) in skin. (**I** and **J**) Levels of *Il33* (**I**) and *Il13* (**J**) mRNA in skin. Data are shown as mean ± SEM from 3 independent experiments (*n* = 3–7 per group). Statistical analysis was performed using 1-way ANOVA (**A**–**E** and **G**–**J**). *P < 0.05, **P < 0.01, ***P < 0.001, ****P < 0.0001.

To investigate the molecular mechanisms for *S*. *aureus*–induced IL-33 expression in keratinocytes, we infected human keratinocyte cell line, KERTr cells, with *S*. *aureus* and found an incremental expression of IL-33 (Supplemental Figure 8A). We also found that *S*. *aureus* triggered the release of lactate dehydrogenase (LDH; an indicator of cell death) from KERTr cells (Supplemental Figure 8B). Similar results were observed in another human keratinocyte cell line, HaCaT cell, with increased production of IL-33 and LDH during *S*. *aureus* infection (Supplemental Figure 8, C–E). Since peptidoglycan (PGN), one of the major components of the *S*. *aureus* cell wall, is known as a TLR2 agonist and has been shown to induce IL-33 expression in myeloid cells (23), we validated if *Il33* mRNA level was affected in KERTr cells infected with *S*. *aureus* or treated with PGN. Accordingly, we found that only live *S*. *aureus* and PGN increased *Il33* transcripts (Figure 7A). Next, we examined how cell death from live infection could intensify IL-33 and LDH levels in the treatments of KERTr cells with live *S*. *aureus*, heat-killed *S*. *aureus* (HK), or PGN. We found that live *S*. *aureus* caused the greatest increase of IL-33 and LDH in the culture supernatant (Figure 7, B and C). In contrast, only a small amount of IL-33 and LDH was detected in the supernatant of KERTr cells treated with HK *S*. *aureus* or PGN (Figure 7, B and C). Similar results were observed in primary mouse keratinocytes (Figure 7D), suggesting that IL-33 release from keratinocytes was positively correlated with the cell death severity.

PGN has been reported to stimulate inflammatory responses through TLR2 signaling; therefore, we investigated whether TLR2 signaling affects IL-33 expression and cell death in *S*. *aureus*–infected KERTr cells. We found that blocking TLR2 signaling with a TLR2 neutralizing antibody attenuated both IL-33 expression and LDH release in *S*. *aureus*–treated KERTr cells (Figure 7E), suggesting that S. aureus–induced IL-33 expression and cell death in keratinocytes are TLR2 signaling dependent. Next, we generated *Tlr2^–/–^* mice to evaluate the effect of TLR2 signaling on *S*. *aureus*–induced skin inflammation. We observed that, in *Tlr2^–/–^* mice, *S*. *aureus* infection did not affect epidermal thickening but did alleviate TEWL and *Il33* expression, and it partially reduced *Il13* expression (Figure 7, F–J). Moreover, the number of infiltrating leukocytes was also reduced in *Tlr2^–/–^* mice compared with WT mice (Supplemental Figure 9). Taken together, these results suggest that *S*. *aureus*–induced IL-33 expression and skin inflammation are dependent on TLR2 signaling.

### S. aureus exacerbates the release of dermal IL-33 through necroptosis.

Previous studies have reported that IL-33, acting as an alarmin, is released upon cell death caused by cellular stress, tissue injury, and infections (24). Therefore, we sought to determine whether the IL-33 release from keratinocytes following *S*. *aureus* infection could be attributed to a specific type of cell death. To ascertain the type of cell death involved in *S*. *aureus* infection, we pretreated KERTr cells with QVD-OPH (a pan-caspase inhibitor), Nec-1 (a RIPK1/RIPK3-mediated necroptosis inhibitor), or NSA (an MLKL-mediated necroptosis inhibitor) before infecting the cells with *S*. *aureus*. All 3 inhibitors suppressed LDH production from 

**Figure 8:**
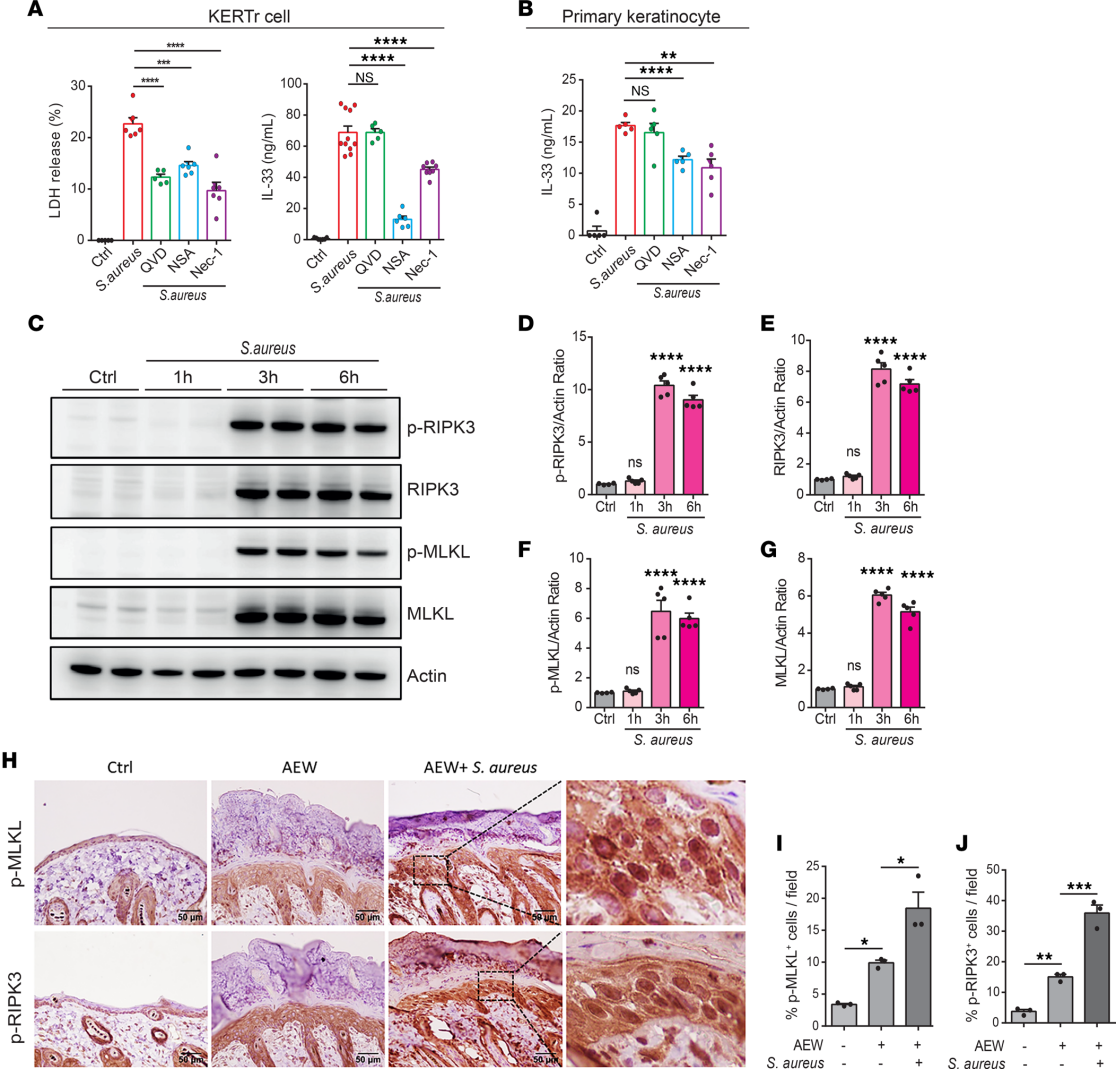
*S*. *aureus*–exacerbated dermal IL-33 release is associated with necroptosis. (**A** and **B**) KERTr cells and mouse primary keratinocytes were pretreated with pancaspase inhibitor QVD-OPH (50 nM), RIPK1 inhibitor Nec-1 (50 nM), or MLKL inhibitor NSA (50 nM) for 1 hour before *S*. *aureus* infection. (**A**) Percentage of LDH and level of IL-33 released from KERTr cells. (**B**) Level of IL-33 released from mouse primary keratinocytes. (**C**) Representative Western blot image of protein expression of p-RIPK3 (54 kDa), RIPK3 (54 kDa), p-MLKL (54 kDa), MLKL (54 kDa), and actin (42 kDa) as control after time-dependent *S*. *aureus* infection of KERTr cells. (**D** and **E**) Quantification of p-RIPK3 and total RIPK3 normalized to actin. (**F** and **G**) Quantification of p-MLKL and total MLKL normalized to actin. (**H**) IHC staining for p-MLKL and p-RIPK3 in the dorsal skin of WT mice. Scale bars: 50 μm. (**I** and **J**) Percentage of p-MLKL^+^ (**I**) and p-RIPK3^+^ keratinocytes (**J**) in the skin. Data are shown as mean ± SEM from 3 independent experiments (*n* = 3–10 per group). Statistical analysis was performed using 1-way ANOVA (**A**, **B**, **D**–**G**, **I**, and **J**). *P < 0.05, **P < 0.01, ***P < 0.001, ****P < 0.0001.

*S. aureus*–infected KERTr cells, suggesting that the infection induces multiple forms of cell death in keratinocytes (Figure 8A). Notably, only the Nec-1 and NSA treatments significantly reduced IL-33 release from the infected keratinocytes, indicating that IL-33 release is specifically associated with necroptotic cell death rather than caspase-mediated cell death (Figure 8A). This observation was confirmed in primary mouse keratinocytes, underscoring the role of necroptosis-mediated signaling in the IL-33 release from *S*. *aureus*–infected keratinocytes (Figure 8B).

Given that the necroptosis pathway is triggered by RIPK3 activation and subsequent RIPK3-mediated MLKL phosphorylation (19), we assessed the expression of phosphorylated MLKL and its upstream effector, phosphorylated RIPK3, in *S*. *aureus*–infected KERTr cells to confirm the involvement of necroptosis. Our results revealed increased expression and phosphorylation of RIPK3 and MLKL shortly after *S*. *aureus* infection (Figure 8, C–G), further suggesting an association between IL-33 release and necroptosis. In *S*. *aureus*–infected mice, we also observed heightened RIPK3 and MLKL phosphorylation in the skin relative to AEW treatment alone (Figure 8, H–J). To further investigate whether p-MLKL^+^ cells also express IL-33, we performed immunofluorescence staining. We observed that *S*. *aureus* infection–augmented p-MLKL^+^ cells were colocalized with IL-33 (Supplemental Figure 10). Collectively, our findings indicate that the increased dermal IL-33 release caused by *S*. *aureus* was linked to necroptosis.

## Discussion

Our study demonstrates that the manipulation of dry skin components leads to the progression of type 2 skin inflammation. The inflammation associated with this dry skin pathogenesis arises from IL-33 production in keratinocytes, and it is accompanied by leukocyte infiltration and elevated IL-13 production from dermal ILC2s. We also discovered that infection with *S*. *aureus* intensifies skin inflammation by enhancing IL-33 release from keratinocytes, and this is dependent on both TLR2 signaling and the necroptotic pathway.

TSLP can activate ILC2s to promote skin inflammation independently of IL-33 (16). To address the role of ILC2s in skin inflammation, the vitamin D3 analog MC-903, known to induce TSLP expression, has been employed as a model of AD-like inflammation (16, 25). However, in our model, we did not detect significant TSLP induction after delipidization. Instead, we observed that lipid removal using an organic solvent prompted IL-33 release from keratinocytes, leading to the development of acute skin inflammation.

Epithelial IL-33 plays significant roles in driving AD-like inflammation. While the *Il33^fl/fl^ K14^cre^* mouse model, which focuses on keratinocytes, does not fully replicate the phenotype observed in global IL-33 KO mice (Figure 2), this incongruity suggests that other IL-33–producing cells beyond keratinocytes may play a significant role in skin inflammation. IL-33 can be released not only from keratinocytes but also from immune and stromal cells within the skin microenvironment (26). It has been reported that mast cells can release IL-33, which subsequently regulates IgE-dependent skin inflammation (27). As shown in Figure 2A, we did observe an increased mast cell population after delipidization. This observation aligns with the notion and demands further exploration that mast cells may contribute to the release of IL-33 in response to changes in the skin environment.

Previous research has shown that Th2 cytokines are elevated in barrier-disrupted skin in mouse models as well as in humans exhibiting AD-like symptoms (28). IL-13 is a potent stimulator of dermal inflammation, and its transgenic expression in the skin can lead to an AD-like phenotype. Additionally, keratinocytes express IL-13 receptors, which can be stimulated with IL-13 to enhance IL-6 expression (29). Our results indicated an increase in IL-13–producing dermal ILC2s in the inflamed skin after delipidization. It is plausible that these cells contribute to keratinocyte damage, resulting in enhanced IL-6 production. However, the exact mechanism remains to be elucidated. We also observed that *Il4* expression was affected in the absence of IL-33 signaling (data not shown). Still, whether IL-4 contributes to skin inflammation and the accumulation of dermal ILC2s needs further exploration.

*S. aureus* infection is prevalent in patients with AD and has been positively correlated with disease severity (4). A recent study reported that *S*. *aureus* penetrates the epidermis, reaching the dermis, and triggers the expression of Th2 cytokines, including IL-4, IL-13, and TSLP, contributing to the exacerbation of the disease (30). These cytokines have been shown to induce keratinocyte cell death through STAT6-mediated signaling (31). Moreover, infection with *S*. *aureus* reportedly elevates IL-22–derived γδ T cells in mechanically injured skin (32), and these cells have been identified as protective against *S*. *aureus* reinfection by producing TNF-α and IFN-γ via the TLR2/MyD88 signaling pathway (33). Moreover, *S*. *aureus* is capable of secreting virulence factors, including biofilm, enterotoxins, and superantigens, that are considered as important elements of the vicious cycle of AD (34). For example, staphylococcal enterotoxin B (SEB) has been reported to elicit IgE antibody production and exacerbate skin inflammation (35). Furthermore, *S*. *aureus*–derived phenol-soluble modulins α (PSMα) have also been identified as inducers of IL-1α and IL-36α release from keratinocytes, thereby contributing to the skin inflammatory response (36). Remarkably, PSMα not only activates and phosphorylates MLKL but also increases the expression and secretion of LDH during *S*. *aureus* infection (37). In our study, we demonstrated that *S*. *aureus* isolated from patients with AD significantly enhanced the expression of IL-33 and IL-6 in KERTr cells. Furthermore, these cytokines promoted the accumulation of IL-13–producing dermal ILC2s and aggravated delipidization-induced AD-like inflammation. In summary, our results offer insights into how *S*. *aureus* infection worsens AD.

Various studies have reported that the maturation and release of IL-33 are mediated by ripoptosome, a protein complex involved in extrinsic apoptotic and necroptotic signaling (38). In an aspergillus asthma disease model, the release of bioactive IL-33 is linked to necroptotic cell death (39). Similarly, RIPK3-induced necroptosis upregulates the *Il33* gene expression in intestinal epithelial cells (40). Accordingly, our study demonstrated that *S*. *aureus* infection induced multiple forms of cell death in keratinocytes. We also confirmed that *S*. *aureus*–exacerbated dermal IL-33 release is necroptosis dependent. While the mechanism behind RIPK3 activation by *S*. *aureus* remains to be elucidated, blocking necroptotic signaling might serve as a therapeutic avenue for AD.

In summary, we demonstrated that delipidization of the skin facilitates the development of AD-like symptoms, characterized by keratinocyte damage and increased IL-33 and Th2 cytokines. Additionally, we found that the microenvironment of delipidized skin encouraged the accumulation of dermal ILC2s, intensifying the pathological symptoms. By mimicking the natural route of *S*. *aureus* infection in patients with AD, our dry-skin mouse model further revealed that the exacerbated IL-33 release from an epicutaneous-infection with *S*. *aureus* was dependent on TLR2 signaling and necroptosis. Our study not only elucidates the mechanism by which *S*. *aureus* infection influences the progression of type 2 inflammation and AD exacerbation in dry skin condition, but it also provides crucial insights for the development of potentially novel interventions to combat AD.

## Methods

### Sex as a biological variable.

Our study examined male and female animals, and similar findings are reported for both sexes.

### Animals.

Three-week-old C57BL/6 and BALB/c mice were purchased from National Laboratory Animal Center. *Rag1^–/–^* and *Rag2^–/–^* mice were purchased from Taconic Farms. *Rag2^–/–^ Il2rg^–/–^*, *Rora^sg/sg^*, *KRT14^cre^*, and *Tlr2^–/–^* mice were purchased from The Jackson Laboratory. *Il13^–/–^* mice were a gift from Andrew J. McKenzie (MRC Laboratory of Molecular Biology, Cambridge, United Kingdom). To generate *Il33^fl/fl^ K14^cre^* mice, *Il-33^fl/fl^* mice were crossed with *KRT14^cre^* mice (purchased from The Jackson Laboratory). *ST2^fl/fl^* mice were crossed with *LysM^cre^* mice (provided by Jr-Wen Shui, Academia Sinica, Taiwan) to generate *ST2^fl/fl^ LysM^cre^* mice. *YetCre-13* mice were crossed with *ROSA^mT/mG^* mice and *ROSA^DTA^* mice (purchased from The Jackson Laboratory) to generate *YetCre-13 ROSA^mT/mG^* mice and *YetCre-13 ROSA^DTA^* mice. All animals were housed under specific pathogen–free conditions.

### Isolation and culture of primary keratinocytes.

For the culture of mouse primary keratinocytes, the skin of neonatal mice was obtained and incubated overnight in 4.3 mg/mL neutral protease at 4°C. The epidermis was then separated from the dermis using forceps and was further digested using 0.25 mg/mL trypsin/EDTA to obtain a single suspension of keratinocytes. Freshly isolated keratinocytes were cultured in antibiotic-free progenitor cell targeted medium (CELLnTEC) in 96-well plates at a density of 1 × 10^5^ cells in 0.2 mL/well.

### Bacterial strains and culture.

*S*. *aureus* (isolated from patients with AD) were provided by Chun-Ming Huang (UCSD, San Diego, California, USA). Strains were cultured in tryptic soy broth (TSB, Sigma-Aldrich) and cultivated overnight at 37°C with 250 rpm gyratory shaking. Aliquots of *S*. *aureus* culture were centrifuged at 2,200*g*; the pellets were washed with phosphate-buffered saline (PBS) and adjusted to an optical density of 1 at 600 nm, corresponding to approximately 1 × 10^9^ *S*. *aureus* cells/mL.

### Delipidization and epicutaneous S. aureus exposure in vivo.

Hair from the dorsal skin (1.0 × 1.0 cm^2^) of mice was shaved before the start of the experiment. For delipidization treatment, the shaved area was treated twice daily with a cotton swab immersed in acetone and ether mixture (1:1) or distilled water for 15 seconds, followed by treatment with distilled water on the same area for 30 seconds, for a duration of either 2 days or 5 days, as previously described (5, 6). Control mice were treated with water. For the *S*. *aureus* infection, 5 × 10^8^ CFU of S. aureus, diluted in 100 μL TSB, were applied onto a cotton pad (1.0 × 1.0 cm^2^). This pad was then placed on the dorsal skin of the mice using a bandage immediately after the last AEW treatment. For the control mice, a pad soaked in TSB alone was applied using a bandage. Mice will be sacrificed 24 hours after the *S*. *aureus* infection.

### Infection with S. aureus in vitro.

KERTr and HaCaT cells were purchased from the American Type Culture Collection and cultured in antibiotic-free keratinocyte-SFM medium (Thermo Fisher Scientific) at 1 × 10^5^ cells/mL in 96-well plates and infected with *S*. *aureus* at a multiplicity of infection (MOI) of 10 for 24 hours, and the supernatant was collected for ELISA measurement. Similar experiments were performed in mouse primary keratinocytes. For mRNA detection, KERTr and HaCaT cells were cultured in antibiotic-free medium at 5 × 10^5^ cells/mL in 12-well plates and infected with *S*. *aureus* at a MOI of 10 for 6 hours.

### Epidermal thickness measurement and histology.

The dorsal skin of mice was excised and fixed with 4% formaldehyde (Merck) for 24 hours and was then gradually dehydrated with 50%, 70%, and 90% ethanol (J.T.Baker) for 15 minutes each. The skin samples were embedded in paraffin for further histological analysis. For measuring epidermal thickness, the skin sections were stained with H&E and observed using an Olympus CX31 microscope (Olympus Corp.).

### Measurement of TEWL.

TEWL was measured using the Dermalab Combo system (C40000.03-189, Cortex Technology) on the dorsal skin of the mice. A probe placed on the skin was stabilized for approximately 30 seconds. Each measurement was performed in triplicate and then averaged.

### Skin processing and cell suspension preparation.

Dorsal skin (1.0 × 1.0 cm^2^) was excised from delipidized mice, cut into smaller pieces with surgical scissors, and incubated in 5 mL DMEM containing 1 mg/mL DNase I (Worthington Biochemicals), 0.5 mg/mL collagenase type I (Worthington Biochemicals), and 0.2 mg/mL collagenase type II (Worthington Biochemicals) for 60 minutes at 37°C. Tissues were filtered through a 70 μm mesh to obtain single-cell suspensions. RBCs were lysed using ACK lysing buffer (Thermo Fisher Scientific), and single-cell suspensions were resuspended in the appropriate buffer for further processing.

### Flow cytometry.

The antibodies used for flow cytometry analysis are listed in Supplemental Table 3. For surface staining, single-cell suspensions were first stained with the fixable viability dye eFluor 455UV (BioLegend). Fc receptors were blocked with anti–mouse CD16/32 (1:100 dilution, TruStain fcX, BioLegend) or anti–human CD16/32/64, and cells were further stained with appropriate antibodies. For intracellular staining, single-cell suspensions were stimulated with 50 ng/mL phorbol 12-myristate 13-acetate (Sigma-Aldrich), 1 μg/mL ionomycin (Sigma-Aldrich), and 1 μg/mL Brefeldin A (BD Biosciences) for 5 hours. After surface staining, cells were fixed and permeabilized with Cytofix/Cytoperm solution (BD Biosciences) and were further stained intracellularly with anti–IL-13 (eBio13A), –IL-5 (TRFK5), –IL-4 (BVD6-24G2), and –IL-9 (RM9A4). After staining, the cells were washed and resuspended in FACS buffer and analyzed using flow cytometry. Data were acquired using an LSR II (BD Biosciences) and analyzed using FlowJo v.10.1 software (Tree Star Inc.). To measure leukocyte infiltration, the frequency of CD45^+^ cells was obtained from flow cytometry analysis and was used to calculate the total number of infiltrated leukocytes.

### Skin draining lymph node dissociation and single-cell isolation.

Peripheral skin-draining lymph nodes (inguinal) were excised from *S*. *aureus*–infected mice. Each draining lymph node was placed in 4.5 mL DMEM, containing 50 μg/mL DNase I and 1 mg/mL collagenase type IV. After 15 minutes at 37°C, DNase and collagenase were neutralized with 10 mM EDTA. Samples were filtered through a 70 μm cell strainer to obtain single-cell suspensions. RBCs were lysed with ACK lysing buffer, and single-cell suspensions were resuspended in the appropriate buffer for further processing.

### Mass cytometry (CyTOF) analysis.

Following skin draining lymph node digestion, single-cell suspensions were washed once with cell staining media (CSM, PBS with 0.5% BSA and 0.02% sodium azide). Fc receptors were blocked with anti–mouse CD16/32 (1:100 dilution, TruStain fcX, BioLegend), and cells were stained with a surface antibody cocktail for 1 hour (Supplemental Table 4). Cells were then washed with CSM and stained with cisplatin (Merck) at a final concentration of 25 μM for 1 minutes at room temperature to label dead cells and then quenched by adding an equal volume of complete medium. Cells were fixed and permeabilized using the Foxp3 transcription factor staining kit (eBioscience) and were then stained with an intracellular antibody cocktail (Supplemental Table 4). Cells were washed twice with CSM and stained for DNA with Cell-ID Intercalator-Ir (^191^Ir and ^193^Ir; Fluidigm). Samples were resuspended in Milli-Q (MilliporeSigma) water, containing EQ Four Element Calibration Beads (Fluidigm) for normalization. Sample acquisition was performed using a CyTOF2 instrument (Fluidigm). Raw flow cytometry standard files, acquired from the CyTOF2 machine, were normalized using the Fluidigm Helios software. Normalized data were analyzed and visualized using viSNE and FlowSOM (Cytobank, Merck). Statistical significance between the 2 groups was determined using the Student’s 2-tailed unpaired *t* test. The gating strategies used for CyTOF analysis are listed in Supplemental Table 2.

### IF and IHC staining of skin sections.

Mouse dorsal skin was harvested and fixed with 4% formaldehyde (Merck) and gradually dehydrated for 15 minutes each with 50%, 70%, and 90% ethanol (J.T.Baker). Paraffin-embedded skin sections were stained using H&E to determine skin thickness. After deparaffinizing and rehydrating, antigen will be retrieved by using Tris-EDTA. For IF staining, skin sections were stained with primary antibodies (Supplemental Table 3), followed by the Alexa Fluor–conjugated secondary antibody (Thermo Fisher Scientific). Slides were incubated with 1% Sudan black (Sigma-Aldrich) for 20 minutes to reduce tissue autofluorescence before mounting. IHC staining will be performed according to the manufacturer’s instructions. Image acquisition was performed using an Olympus BX51 microscope.

### ELISA.

The supernatants from the KERTr, HaCaT, and primary keratinocytes were collected and assayed for cytokines by using ELISA kits according to the manufacturer’s instructions. The ELISA kits used for cytokine detection are listed in Supplemental Table 5.

### LDH.

The supernatants from KERTr, HaCaT, and primary keratinocytes were collected and assayed to determine LDH levels using Cytotoxicity Detection Kit (Supplemental Table 5) according to the manufacturer’s instructions.

### Immunoblotting.

KERTr cells (2 × 10^6^ cells/well) were lysed in protein lysis buffer, sonicated, and centrifuged at 13,000*g* for 10 minutes at 4°C to remove cell debris. Protein concentration was determined using the micro-BCA protein assay kit (Thermo Fisher Scientific). Equal amounts of protein (12 μg/sample) were separated using SDS-PAGE and transferred to PVDF membranes (PALL Corporation). Membranes were blocked with Tris-buffered saline and Tween 20 containing 5% BSA. The primary antibodies for immunoblotting are listed in Supplemental Table 3. The primary antibodies were detected with peroxidase-conjugated anti–rabbit IgG or anti–mouse IgG, followed by detection with Western Lightning ECL Pro kit (PerkinElmer) according to the manufacturer’s recommendations (PerkinElmer, NEL120E001EA).

### Quantitative PCR.

Total RNA was isolated from skin tissue or cultured cells and extracted using the Quick-RNA MicroPrep (Zymo Research) according to the manufacturer’s instructions. In total, 1–2 μg of cDNA was synthesized using the high-capacity cDNA reverse transcription kit (Applied Biosystems), and real-time PCR was conducted using an Optical 96 real-time PCR thermal cycler (Biometra). Reactions were run in triplicate, samples were normalized to *GAPDH* expression, and quantities were determined according to the 2^–ΔΔCt^ method. The primers used for quantitative PCR (qPCR) are listed in Supplemental Table 6.

### Statistics.

Statistical analyses were performed using Prism 6 (GraphPad Prism). Student’s 2-tailed unpaired *t* test or 1-way ANOVA were used to determine statistical significance between groups; *P* < 0.05 was considered significant.

### Study approval.

All animal use procedures were approved by the Academia Sinica IACUC (protocol ID: 1412764), and all experiments were performed according to the guidelines of IACUC.

### Data availability.

Values for all data points in graphs are reported in the Supporting Data Values file.

## Author contributions

YJC initiated and designed the study. CHL, CCT, WYC, YSC, and EJCC conducted experiments and analyzed the data. CHL, ACYL, CCT, and YJC wrote the manuscript. CHL, ACYL, and YJC reviewed and edited the manuscript.

## Supplementary Material

Supplemental data

Unedited blot and gel images

Supporting data values

## Version 1 02/06/2024

In-Press Preview

## Version 2 03/22/2024

Electronic publication

## Version 3 08/27/2024

Figures 7 and 8 were accidentally swapped. Also, Figure 8H is updated
