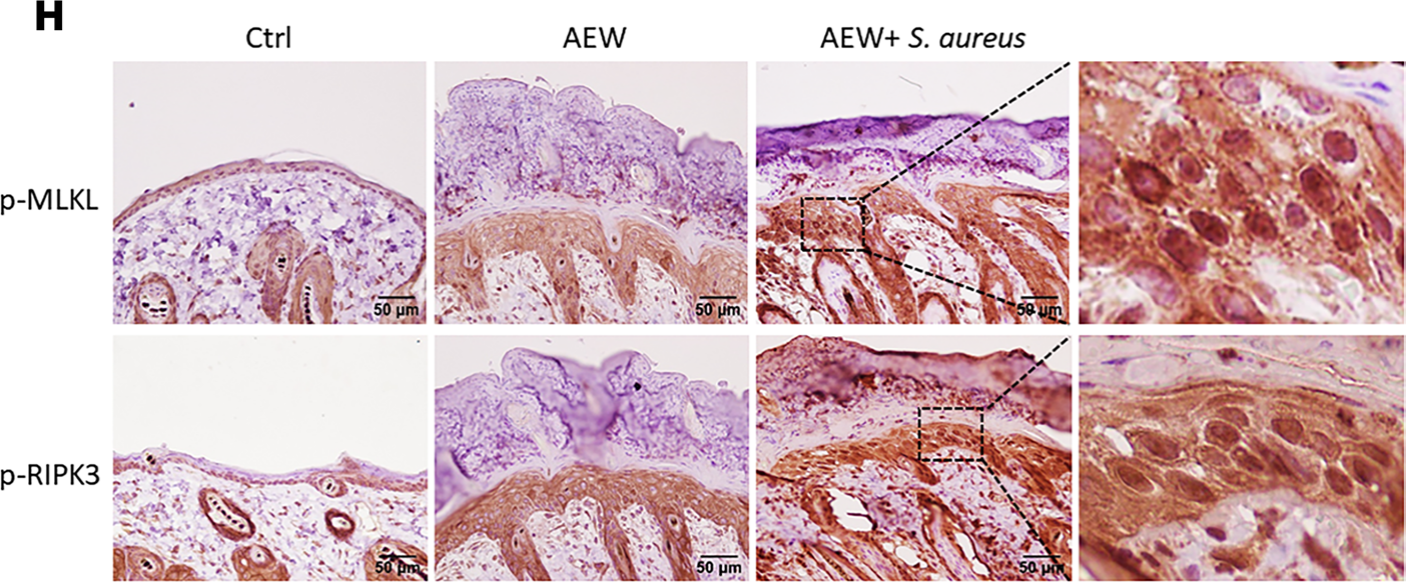# *Staphylococcus aureus* exacerbates dermal IL-33/ILC2 axis activation through evoking RIPK3/MLKL-mediated necroptosis of dry skin

**DOI:** 10.1172/jci.insight.185878

**Published:** 2024-09-10

**Authors:** Chia-Hui Luo, Alan Chuan-Ying Lai, Chun-Chou Tsai, Wei-Yu Chen, Yu-Shan Chang, Ethan Ja-Chen Chung, Ya-Jen Chang

Original citation *JCI Insight*. 2024;9(6):e166821. https://doi.org/10.1172/jci.insight.166821

Citation for this corrigendum: *JCI Insight*. 2024;9(17):e185878. https://doi.org/10.1172/jci.insight.185878

The authors recently became aware that the p-RIPK3–stained control panel presented in Figure 8H was incorrect. The correct figure panel is shown below. In addition, during the preparation of the manuscript for publication, Figures 7 and 8 were inadvertently swapped. The HTML and PDF files have been updated.

The authors and the *JCI* regret the errors. 

## Figures and Tables

**Figure 8:**