# Supplementary material for: Staphylococcus aureus exacerbates dermal IL-33/ILC2 axis activation through evoking RIPK3/MLKL-mediated necroptosis of dry skin
Source: JCI Insight. 2024 Feb 6;9(6):e166821. doi: 10.1172/jci.insight.166821 (PMC11063943; doi:10.1172/jci.insight.166821)
Supplement: Supplemental data [file jciinsight-9-166821-s009.pdf]

ONLINE SUPPLEMENTARY MATERIAL

Original Article

Staphylococcus aureus exacerbates dermal IL-33-ILC2 axis activation through evoking RIPK3/MLKL-mediated necroptosis of dry skin

Chia-Hui Luo[1,2], Alan Chuan-Ying Lai[1], Chun-Chou Tsai[1], Wei-Yu Chen[3], Yu-Shan Chang[1], Ethan Ja-Chen Chung[1], Ya-Jen Chang[1,4,5,6,#]

[1] Institute of Biomedical Sciences, Academia Sinica, Taipei, Taiwan

[2] Taiwan International Graduate Program in Molecular Medicine, National Yang Ming Chiao Tung University and Academia Sinica, Taipei, Taiwan

[3] Department of Biochemistry and Molecular Biology, National Cheng Kung University, Tainan, Taiwan

[4] Institute of Microbiology and Immunology, National Defense University, Taipei, Taiwan

[5] Institute of Translational Medicine and New Drug Development, China Medical University, Taichung, Taiwan

[6] Graduate Institute of Medicine, College of Medicine, Kaohsiung Medical University, Kaohsiung, Taiwan

#Corresponding authors: Ya-Jen Chang, Ph.D., E-mail: yajchang@ibms.sinica.edu.tw, Tel: +886-2-27899050.

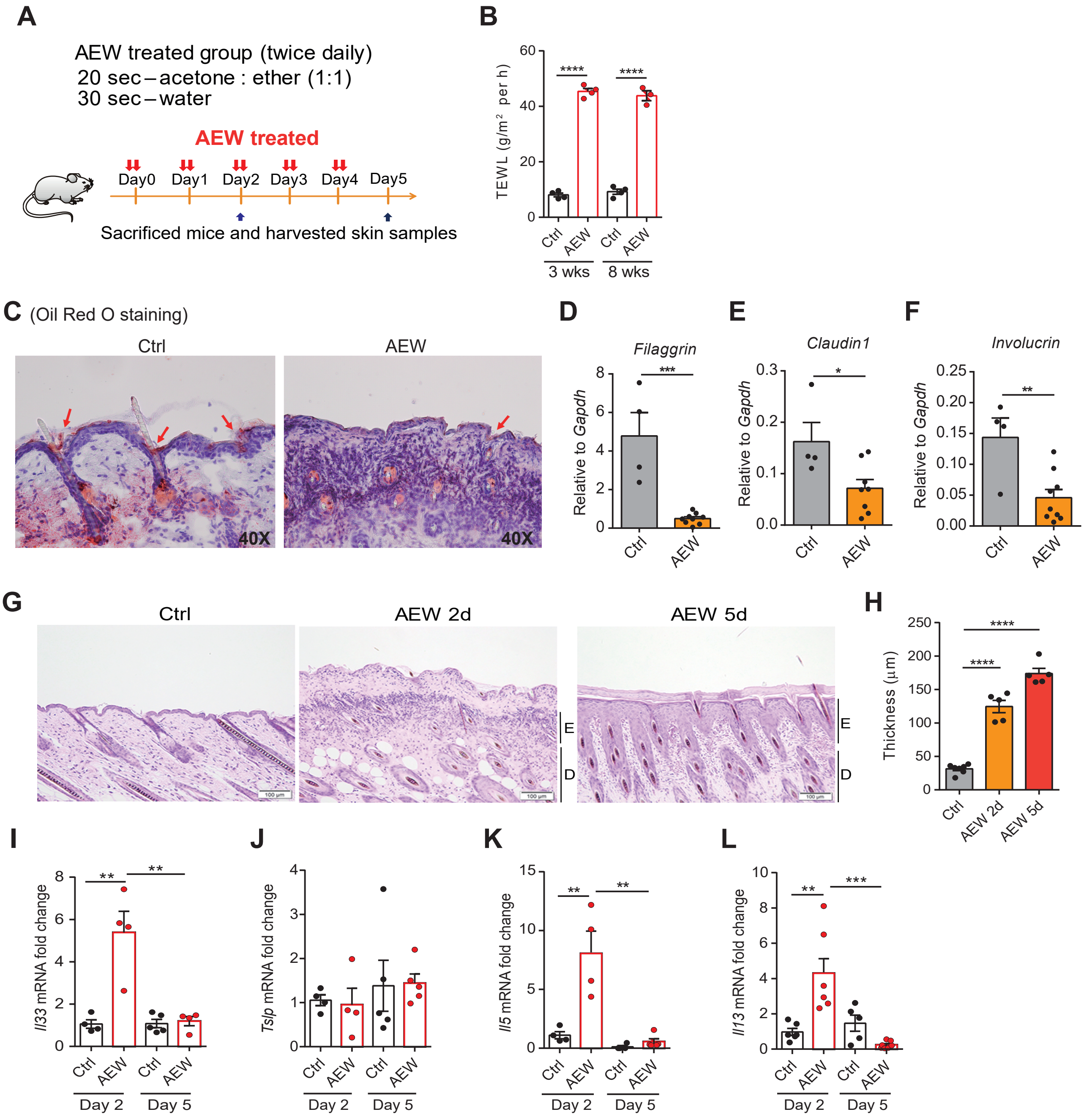

Supplementary Figure 1. Delipidization affects transepidermal water loss, skin thickness, and skin integrity.

(**A**) Schematic diagram of the AEW protocol. (**B-L**) Mice were treated with AEW (twice daily) for 2 days or 5 days and were sacrificed one day after the last treatment; controls were treated with water. (**B**) Transepidermal water loss (TEWL) in delipidized skin. (**C**) Oil Red O staining in skin sections. (Scale bars: 50 µm, Magnification: 40X). Red arrows indicate lipid component. (**D-F**) mRNA levels of (**D**) *Filaggrin*, (**E**) *Claudin1*, and (**F**) *Involucrin* in the skin. (**G**) H&E-stained skin sections of mice treated with AEW or not for 2 and 5 days. (Scale bars: 100 µm,). (E: epidermidis; D: dermal). (**H**) Measurement of skin thickness. (**I-L**) mRNA levels of (**I**) *Il33*, (**J**) *Tslp*, (**K**) *Il5*, and (**L**) *Il13* in the skin. Data are shown as mean ± SEM from 3 independent experiments (n=4-8 per group). Statistical analysis was performed using one-way ANOVA (**B**, **H**, and **I-L**) or an unpaired two-tailed t test (**D-F**). *$*p<0.05$, $**p<0.01$, $***p<0.001$, $****p<0.0001$.*

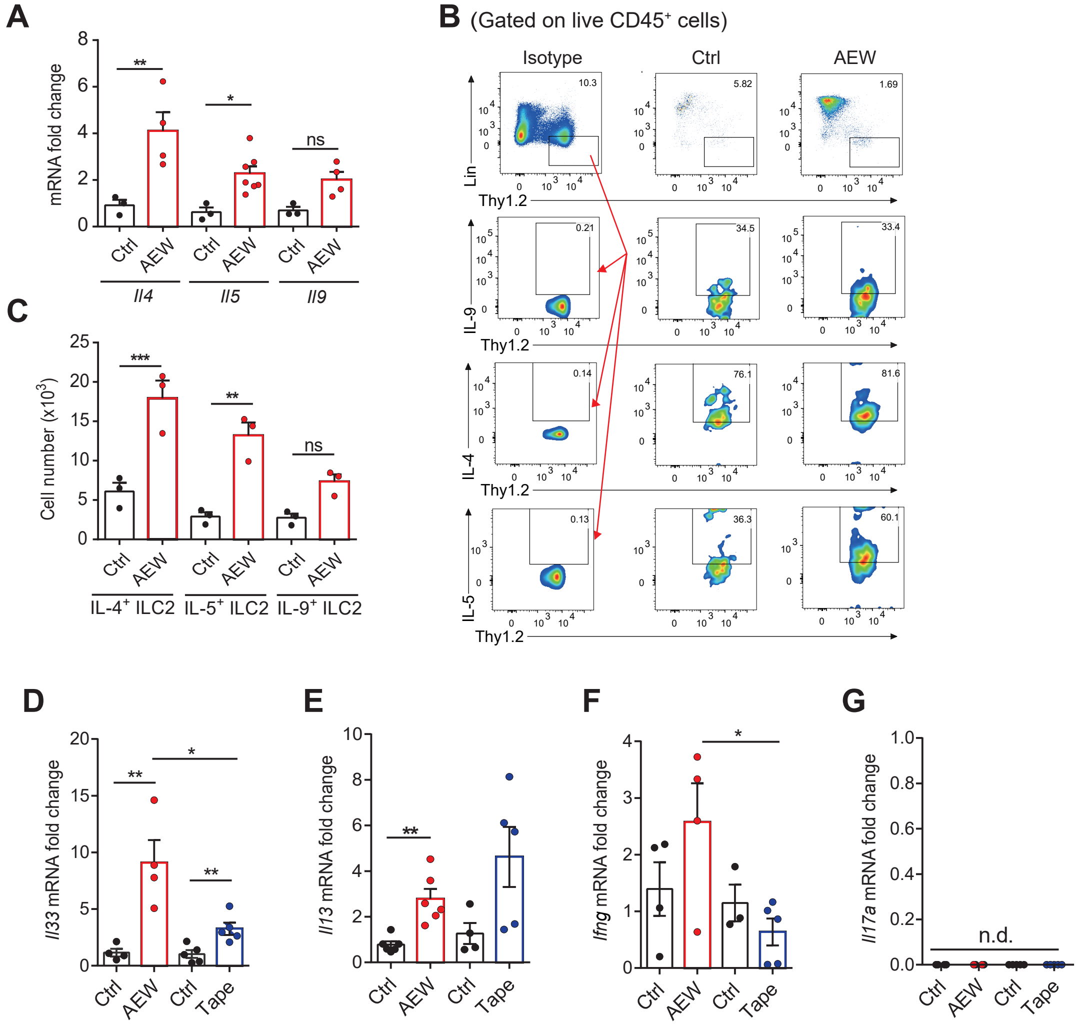

Supplementary Figure 2. Delipidization increases IL-4 and IL-5 expression in the skin, and AEW treatment induces higher IL-33 expression compared to tape stripping treatment.

(**A-G**) Mice were treated with AEW (twice daily) for 2 days and were sacrificed one day after the last treatment. (**A**) mRNA levels of *Il4*, *Il5*, and *Il9* in the skin. (**B**) Representative FACS analysis of IL-9, IL-4, and IL-5 expressions in dermal ILC2s (CD45$^+$Lin$^-$Thy1.2$^+$). (**C**) Total number of IL-4$^+$, IL-5$^+$, and IL-9$^+$ dermal ILC2s in the skin. (**D-G**) Three-week-old WT mice were treated with AEW or tape-stripped for 2 days, twice daily, and sacrificed one day after the last treatment. mRNA levels of (**D**) *Il33*, (**E**) *Il13*, (**F**) *Ifng*, and (**G**) *Il17a* in skin lesions. Data are shown as mean ± SEM from 3 independent experiments (n=3-7 per group). Statistical analysis was performed using one-way ANOVA (**A**, **C**, and **D-G**). *n.s.* Not significant. *n.d.* Not detectable. **p<0.05, **p<0.01, ***p<0.001.*

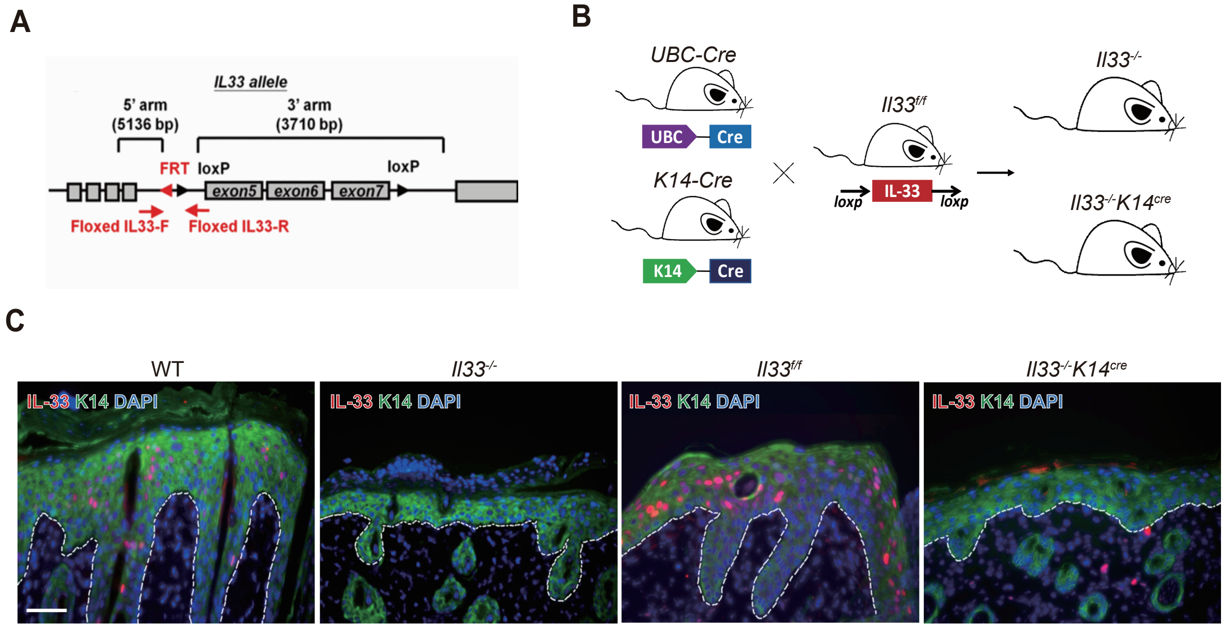

Supplementary Figure 3. Generation and validation of IL-33 conditional knockout mice.

(**A**) Schematic diagram showing Il33 floxed and deleted loci. Exons 5, 6, and 7 of Il33 were flanked by two LoxP sites (arrowheads). (**B**) *Il33*$^{f/f}$ mice were crossbred with mice carrying UBC-Cre and/or K14-Cre to obtain *Il33*$^{-/-}$ mice and/or conditional knockout *Il33*$^{f/f}$*K14*^{cre} mice. (**C**) Immunofluorescence staining of IL-33 (red), K14 (green), and DAPI (blue) in the skin of the indicated mice. (Scale bars: 100 μm).

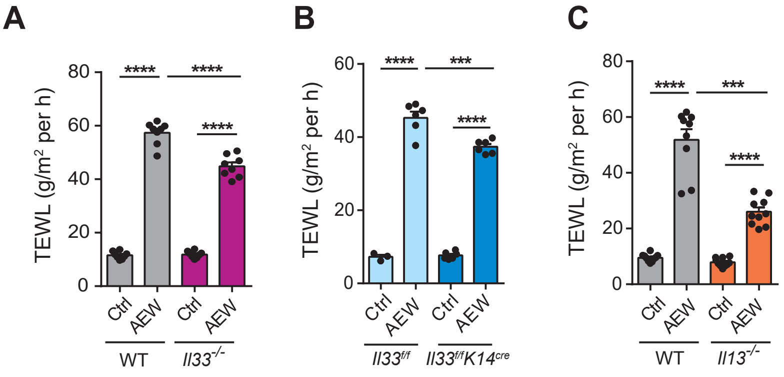

Supplementary Figure 4. Transepidermal water loss (TEWL) in *Il33*$^{-/-}$, *Il33*$^{f/f}$*K14*^{cre}, and *Il13*$^{-/-}$ mice under delipidization.

(**A-C**) Three-week old mice were treated with AEW for 2 days and were sacrificed one day after the last treatment. (**A**) TEWL in the skin of *Il33*$^{-/-}$ and WT mice. (**B**) TEWL in the skin of *Il33*$^{f/f}$ and *Il33*$^{f/f}$*K14*^{cre} mice. (**C**) TEWL in the skin of *Il13*$^{-/-}$ and WT mice. Data are shown as mean ± SEM from 3 independent experiments (n=4-9 per group). Statistical analysis was performed using one-way ANOVA. ****p<0.001,* *****p<0.0001.*

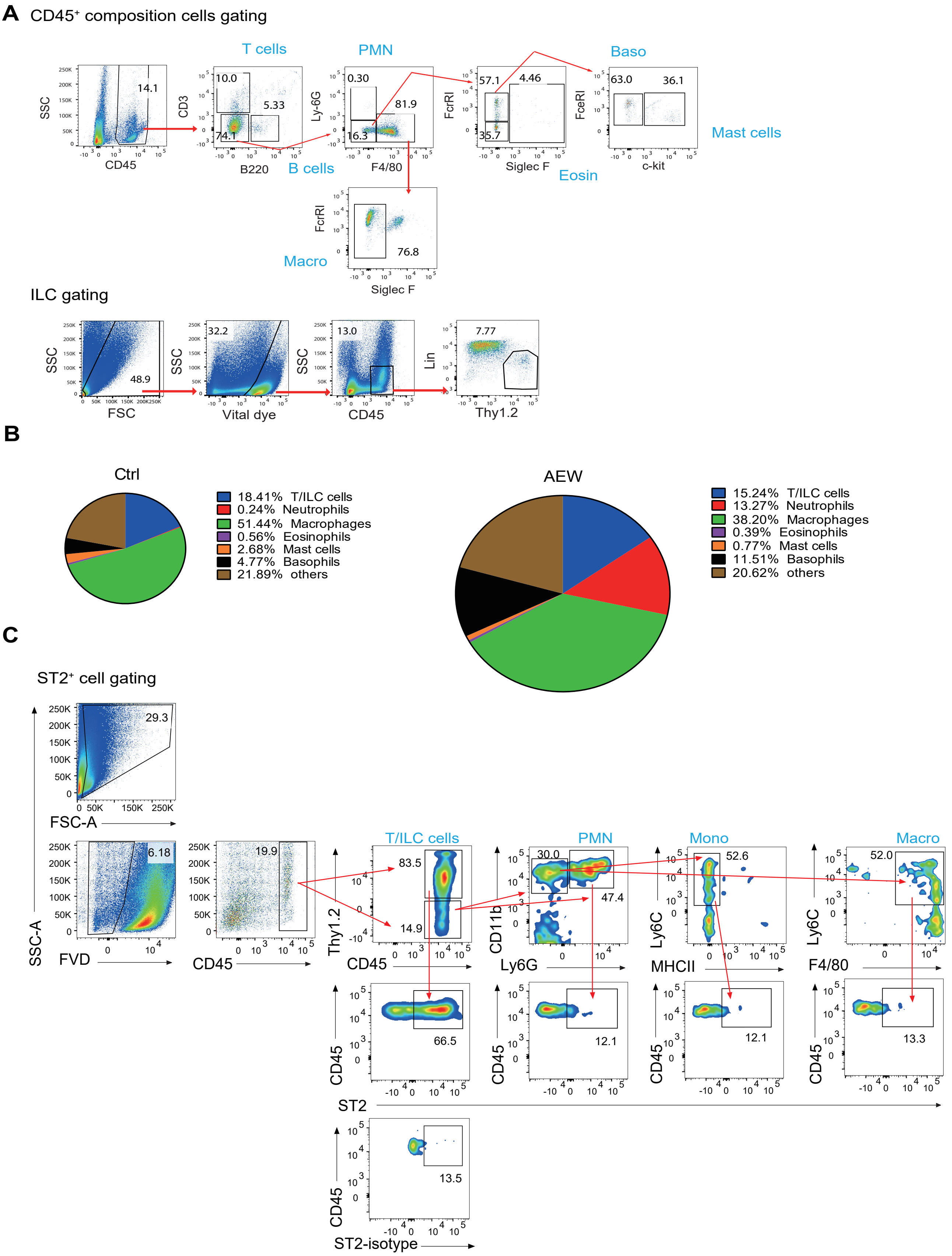

Supplementary Figure 5. Gating strategy of dermal leukocytes, innate lymphoid cells, and ST2$^+$ leukocytes following delipidization treatment.

(**A-C**) C57BL/6 mice were subjected to a 2-day treatment with AEW and sacrificed one day after the last treatment. (**A**) Gating strategies for dermal T cells (CD45$^+$ CD3$^+$), B cells (CD45$^+$ B220$^+$), neutrophils (PMN, CD45$^+$ Ly6G$^+$), macrophages (macro, CD45$^+$ F4/80$^+$ FceRI$^+$), eosinophils (eosin, CD45$^+$ FceRI$^{-/+}$ SiglecF$^+$), basophils (baso, CD45$^+$ FceRI$^+$ c-kit), and mast cells (CD45$^+$ FceRI$^+$ c-kit$^+$) and for dermal ILCs (CD45$^+$ Lin$^-$Thy1.2$^+$). (**B**) Pie charts depicting the relative proportions of lymphocytes and myeloid cells among the CD45$^+$ cells present in the skin. (**C**) Gating strategy for dermal ST2$^+$ leukocytes, including T/ILC cells (CD45$^+$ Th1.2$^+$ ST2$^+$), neutrophils (PMN, CD45$^+$ CD11b$^+$ Ly6G$^+$ ST2$^+$), monocytes (mono, CD45$^+$ CD11b$^+$ Ly-6C$^+$ MHCII$^-$ ST2$^+$), and macrophages (macro, CD45$^+$ CD11b$^+$ Ly6C$^+$ F4/80$^+$ ST2$^+$). All of the cells were gated from live cells.

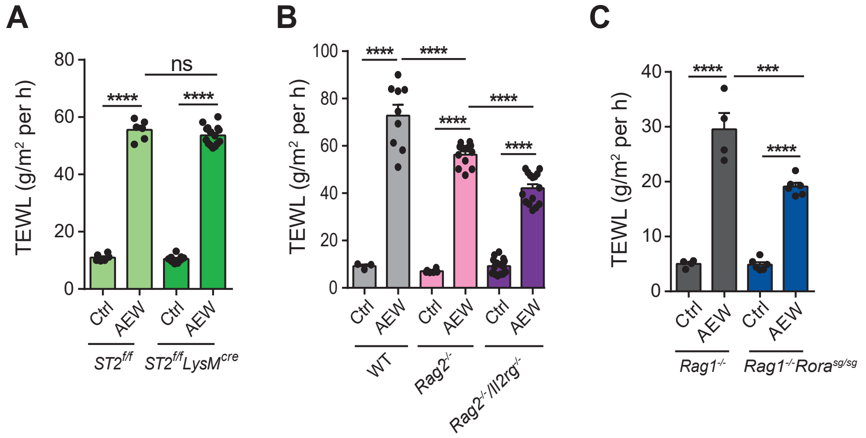

Supplementary Figure 6. Transepidermal water loss (TEWL) in various KO mice under delipidization.

(**A-C**) Three-week old mice were treated with AEW for 2 days and were sacrificed one day after the last treatment. (**A**) TEWL in the skin of *ST2*$^{f/f}$ and *ST2*$^{f/f}$*LysM*^{cre} mice. (**B**) TEWL in the skin of *Rag2*$^{-/-}$ and *Rag2*$^{-/-}$/*Il2rg*$^{-/-}$ mice. (**C**) TEWL in the skin of *Rag1*$^{-/-}$ and *Rag1*$^{-/-}$*Rora*$^{sg/sg}$ mice. Data are shown as mean ± SEM from 3 independent experiments (n=4-9 per group). Statistical analysis was performed using one-way ANOVA. *n.s.* Not significant. ****p<0.001, ****p<0.0001.*

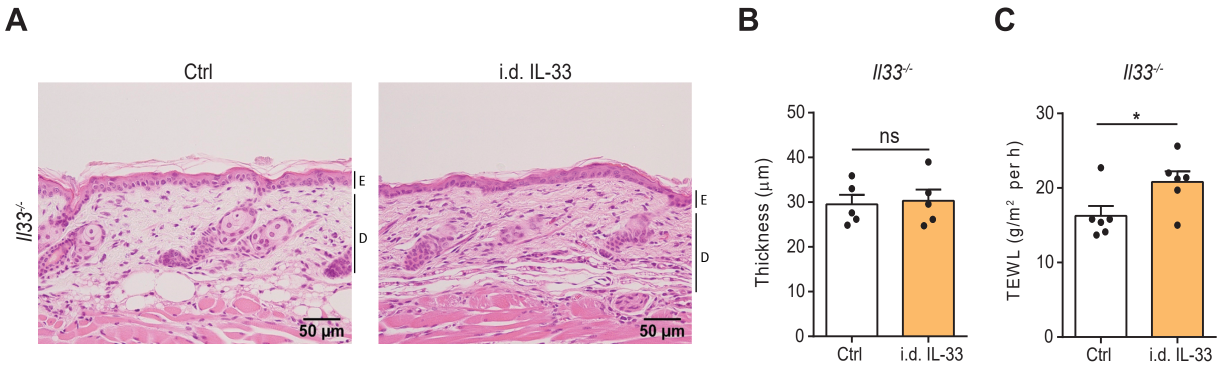

Supplementary Figure 7. Epidermal thickness and TEWL in the skin of *Il33*$^{-/-}$ mice.

(**A-C**) Three-week-old *Il33*$^{-/-}$ mice were administered 1 µg of IL-33 recombinant proteins intradermally (*i.d.*) once daily for three days and were sacrificed one day after the last treatment; controls received vehicle. (**A**) H&E-stained skin sections. (Scale bars: 50 µm, Magnification: 40X). (E: epidermidis; D: dermal). (**B**) Measurement of skin thickness. (**C**) TEWL in the skin of *Il33*$^{-/-}$ mice. Data are shown as mean ± SEM from 3 independent experiments (n=4-9 per group). Statistical analysis was performed using unpaired two-tailed t test (**B-C**). *n.s.* Not significant. *$*p<0.05$.*

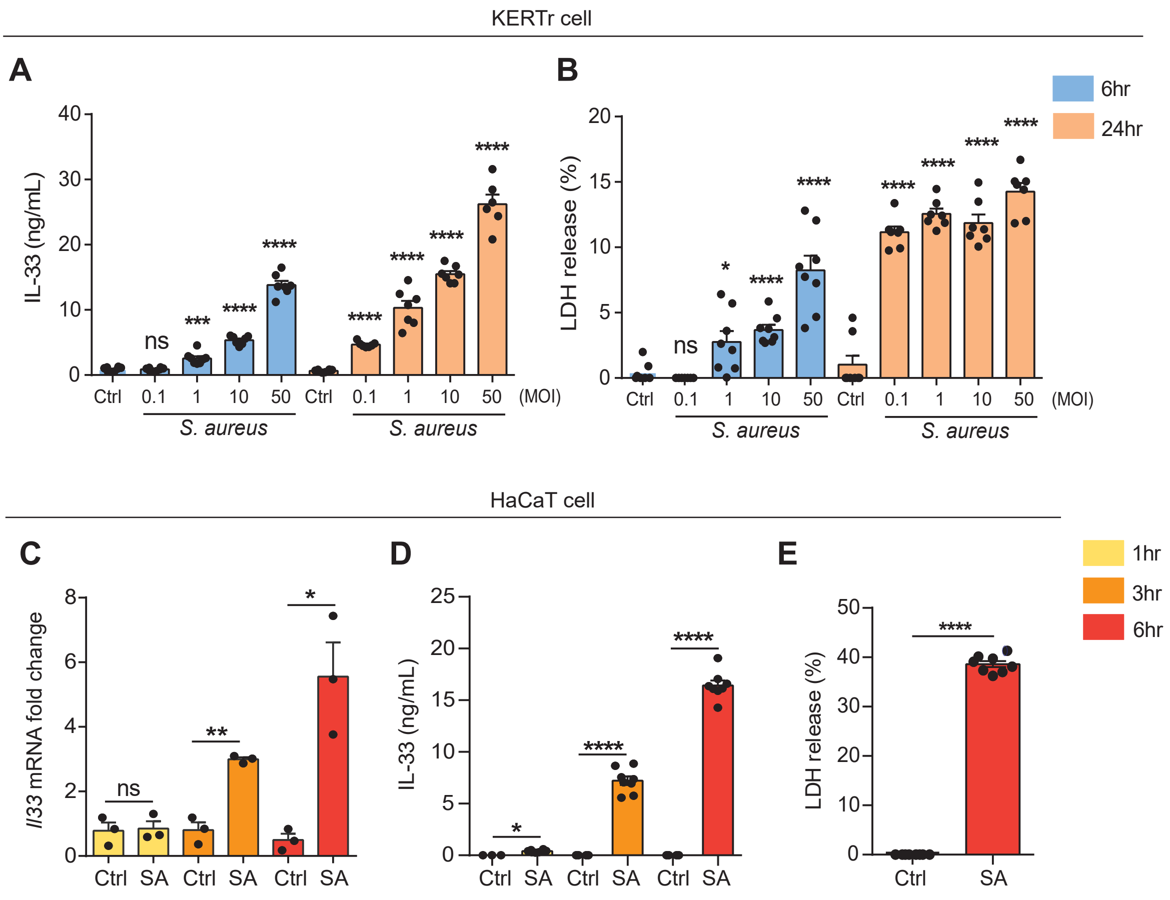

Supplementary Figure 8. *S. aureus* increases IL-33 expression and LDH release in an infection dose and time-dependent manner in KERTr cells and HaCaT cells.

(**A-B**) (**A**) IL-33 levels in the culture supernatant and (**B**) the percentage of LDH released from KERTr cells infected with *S. aureus* (SA) at the different multiplicity of infection (MOI) for 6 h and 24 h. (**C-D**) IL-33 expression levels from HaCaT cells infected with *S. aureus* for 1 h, 3 h, and 6 h. (**E**) Percentage of LDH released from HaCaT cells infected with *S. aureus* for 6 h. Data are shown as mean ± SEM from 3 independent experiments (n=3-8 per group). Statistical analysis was performed using one-way ANOVA (**A-D**) or an unpaired two-tailed t test (**E**). *n.s.* Not significant. * *p<0.05*, ** *p<0.01*, *** *p<0.001*, ****$p< 0.0001$.

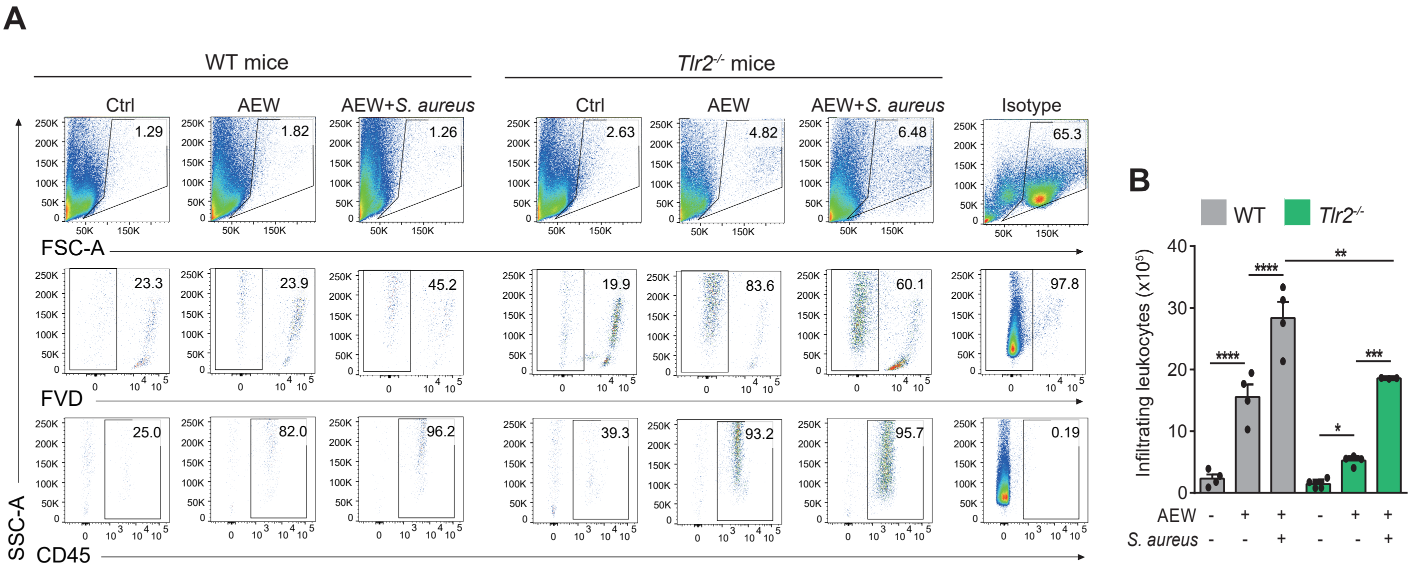

Supplementary Figure 9. ***S. aureus*****-induced leukocyte infiltration is dependent on TLR2 signaling.**

(**A-B**) Three-week-old C57BL/6 and *Tlr2*$^{-/-}$ mice were epicutaneously challenged with *S. aureus* (5× 10^8 CFU) under delipidization treatment for 24 h. (**A**) Representative FACS analysis of CD45$^+$ leukocytes in the skin. (**B**) Numbers of CD45$^+$ leukocytes in the skin. Data are shown as mean ± SEM from 3 independent experiments (n=4-6 per group). Statistical analysis was performed using one-way ANOVA (**B**). * $p<0.05$, ** $p<0.01$, *** $p<0.001$, ****$p< 0.0001$.

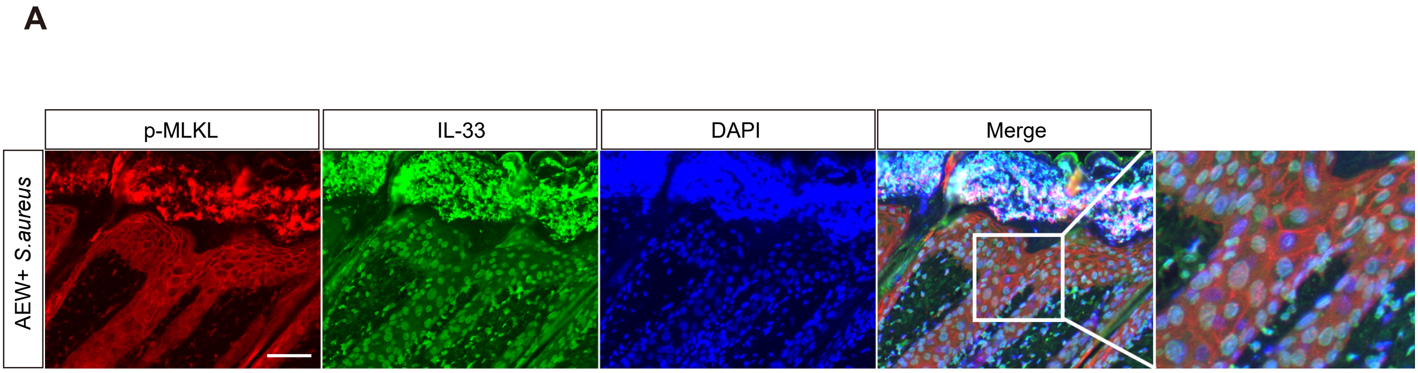

Supplementary Figure 10. ***S. aureus*** **infection-augmented p-MLKL⁺ cells are co-localized with IL-33.**

(**A**) Image of immunofluorescence staining in mice skin. (Scale bars: 50 μm).

Supplementary Tables

Table S1. Frequencies of CD45^{+} immune cell subsets in *S. aureus* infection under delipidization treatment

**Immune cell subsets**		**IL-33$^{f/f}$**			**IL-33$^{f/f}$K14^{cre}**				***P* value** [a]
		**1**	**2**	**3**	**1**	**2**	**3**	**4**	
**CD45^{+} cells**	B cells	37.84	45.6	47.86	42.56	48.15	50.23	57.08	0.2456
	Dendritic cells	0.43	0.62	0.41	0.57	0.48	0.62	0.34	0.8697
	Macrophages	0.22	1.16	0.52	0.32	0.76	0.7	0.5	0.8175
	CD4 T cells	25.72	16.63	21.97	22.65	13.68	17.92	15.79	0.2696
	CD8 T cells	27.95	20.17	21.15	21.72	17.34	18.61	15.08	0.1205
	ILC2	1.27	1.09	1.81	0.6	0.68	1.36	0.7	0.0288
	Treg	2.13	1.79	1.96	1.51	1.53	1.79	1.45	0.0235
	γδ T cells	0.36	0.29	0.27	0.18	0.18	0.22	0.16	0.0066

[a] n=3-4 per group; statistical significance between the two groups was determined using Student's t-test.

Table S2. Gating strategies used for CyTOF analysis

Immune cell subsets		Gating strategy
$CD45^{+}$ cells	B cells	$CD19^{+}$ $CD90^{-}$
	Dendritic cells	$CD90^{-}$ $F4/80^{-}$ $CD11c^{+}$ $CD11b^{+}$
	Macrophages	$CD90^{-}$ $MHCII^{+}$ $F4/80^{+}$
	CD4 T cells	$CD3^{+}$ $CD4^{+}$ $CD8^{-}$
	CD8 T cells	$CD3^{+}$ $CD4^{-}$ $CD8^{+}$
	ILC2	$CD4^{-}$ $CD90^{+}$ $GATA3^{+}$
	Treg	$CD3^{+}$ $Foxp3^{+}$
	γδ T cells	$CD3^{+}$ $CD4^{-}$ $CD8^{-}$ $TCRd^{+}$

Table S3. Antibodies used for flow cytometry, tissue staining, and immunoblotting.

Antibody/Reagent	Clone	Concentration (μg/ml)	Supplier
PerCP/Cy5.5 anti-CD45	30-F11	FC (0.5)	BioLegend
PE anti-SiglecF	E50-2440	FC (1.0)	BioLegend
FITC anti-CD11b	M1/70	FC (0.5)	BioLegend
BV785 anti-CD3	117A2	FC (0.5)	BioLegend
PE-Cy7 anti-MHCII	M5/114.15.2	FC (1.0)	BioLegend
PE-Cy7 anti-F4/80	BM8	FC (0.5)	BioLegend
FITC anti-FcεRI	MAR-1	FC (0.5)	BioLegend
APC anti-ST2	DIH9	FC (1.0)	BioLegend
BV421 anti-c-Kit	2B8	FC (1.0)	BioLegend
APC-Cy7 anti-Ly6G	1A8	FC (0.5)	BioLegend
BV605 anti-Ly6G	1A8	FC (0.5)	BioLegend
BV605 anti-Thy1.2	53-2.1	FC (0.5)	eBioscience
PE-Cy7 anti-ICOS	c398.4A	FC (1.0)	BioLegend
PE anti-T-bet	eBio4B10	FC (0.5)	eBioscience
BV421 anti-GATA3	16E10A23	FC (0.5)	BioLegend
APC anti-RORγt	AFKJS-9	FC (0.5)	eBioscience
PE anti-CD103	15G10	FC (0.5)	BioLegend
APC anti-B220	RA3-6B2	FC (0.5)	BioLegend
Anti-IL-33	N/A	IF (1:100)	R&D Systems
Anti-K14	N/A	IF (1:200)	Abcam
PE anti-GATA3	TWAJ	IF (1:200)	eBioscience
FITC anti-KLRG1	2F1	IF (1:200)	BioLegend
anti-Actin	N/A	WB (1:2000)	Santa Cruz
anti-RIPK3	N/A	WB (1:2000)	Abcam
anti-phospho-RIPK3	EPR9516(N)-25	WB (1:2000)<br>IHC (1:100)	Abcam
anti-MLKL	N/A	WB (1:2000)	GeneTex
anti-phospho-MLKL	EPR9515(2)	WB (1:2000)<br>IHC (1:100)<br>IF (1:100)	Abcam

Table S4. Antibodies used for CyTOF analysis

Antigen-immune panel	Symbol	Mass	Antibody clone	Brand
CD90	In	113	30-H12	BioLegend
H3K9ac	In	115	C5B11	Cell Signaling
mhcCD44	Cd	116	IM7	BD Biosciences
CD11b	Ce	140	M1/70	BioLegend
CD69	Pr	141	H1.2F3	BioLegend
CD45	Nd	142	30-F11	BioLegend
CD11c	Nd	143	HL3	BD Biosciences
Gr1	Nd	144	RB6-8C5	BioLegend
CD4	Nd	145	RM4-5	Fluidigm
CD38	Nd	146	90	BioLegend
CD3	Sm	147	17A2	BioLegend
CD103	Nd	148	2E7	BioLegend
CD19	Sm	149	6D5	Fluidigm
CD27	Nd	150	LG.3A10	Fluidigm
Ly6C	Eu	151	HK1.4	BioLegend
Ki-67	Sm	152	SolA15	eBioscience
PD-L1	Eu	153	10F.9G2	Fluidigm
Tim-3	Sm	154	RMT3-23	BioLegend
CD8a	Gd	155	53-6.7	BioLegend
Eomes	Gd	156	Dan11mag	eBioscience
Foxp3	Gd	158	FJK-16S	eBioscience
PD-1	Tb	159	29F.1A12	BioLegend
GATA3	Gd	160	TWAJ	eBioscience
Tbet	Dy	161	O4-46	Fluidigm
TCRd	Dy	162	GL3	BioLegend
CD80	Dy	163	16-10A1	BioLegend
CD62L	Dy	164	MEL-14	Fluidigm
NK1.1	Ho	165	PK136	Fluidigm
cKit	Er	166	2B8	BioLegend
NKp46	Er	167	29A1.4	BioLegend
RORγR	Er	168	600214	Fluidigm
F4/80	Tm	169	BM8	BioLegend
CD137(41BB)	Er	170	17B5	BioLegend
CD64	Yb	171	X54-5/7.1	BioLegend
H3K27ac	Yb	172	MABI0309	GeneTex
FceRI	Yb	173	MAR-I	BioLegend
mSiglecF	Yb	174	E50-2440	BD Biosciences
CD127	Lu	175	A7R334	Fluidigm
ST2	Yb	176	DIH9	BioLegend
MHCII	Bi	209	M5/114.15.2	Fluidigm
DNA	Ir	191/193		
Cisplatin Viability	Pt	195		

Table S5. Critical commercial assays

Reagent	Supplier	Identifier
Mouse IL-33 ELISA Kit	R&D	Cat# DY3626-15
Human IL-33 ELISA Kit	PeproTech	Cat# 900-k398
Cytotoxicity Detection Kit (LDH)	Roche	Cat# 4744934001
Immunohistochemistry (IHC) kit	Nichirei Biosciences	Cat# 414351F

Table S6. Primers used for qRT-PCR

Gene	Species	Sequence (5’-3’)
Gapdh	mouse	Forward: AGGTCGGTGTGAACGGATTTG<br>Reverse: TGTAGACCATGTAGTTGAGGTCA
IL6	mouse	Forward: CAAAGCCAGATCAGA<br>Reverse: GATGGTCTTGGTCCTTAGCC
IL33	mouse	Forward: ATTTCCCCGGCAAAGTTCAG<br>Reverse: AACGGAGTCTCATGCAGTAGA
Tslp	mouse	Forward: AGGCTACCCTGAAACTGAG<br>Reverse: GGAGATTGCATGAAGGAATACC
IL13	mouse	Forward: CCTGGCTCTTGCTTGCCTT<br>Reverse: GGTCTTGTGTGATGTTGCTCA
Ifn-γ	mouse	Forward: GGCCATCAGCAACAACATAAGCGT<br>Reverse: TGGGTTGTTGACCTCAAACTTGGC
IL17A	mouse	Forward: TCCAGAAGGCCCTCAGACTA<br>Reverse: ACACCCACCAGCATCTTCTC
Gapdh	human	Forward: AGGTCGGAGTCAACGGATTTG<br>Reverse: TGTAAACCATGTAGTTGAGGTC
IL6	human	Forward: AGCCACTCACCTCTTCAGAACGAA<br>Reverse: AGTGCCTCTTTGCTGCTTTCACAC
IL33	human	Forward: CAAAGAAGTTTGCCCCATGT<br>Reverse: AAGGCAAAGCACTCCACAGT
