# Supplementary material for: Staphylococcus aureus exacerbates dermal IL-33/ILC2 axis activation through evoking RIPK3/MLKL-mediated necroptosis of dry skin
Source: JCI Insight. 2024 Feb 6;9(6):e166821. doi: 10.1172/jci.insight.166821 (PMC11063943; doi:10.1172/jci.insight.166821)
Supplement: Unedited blot and gel images [file jciinsight-9-166821-s010.pptx]

## Slide 1
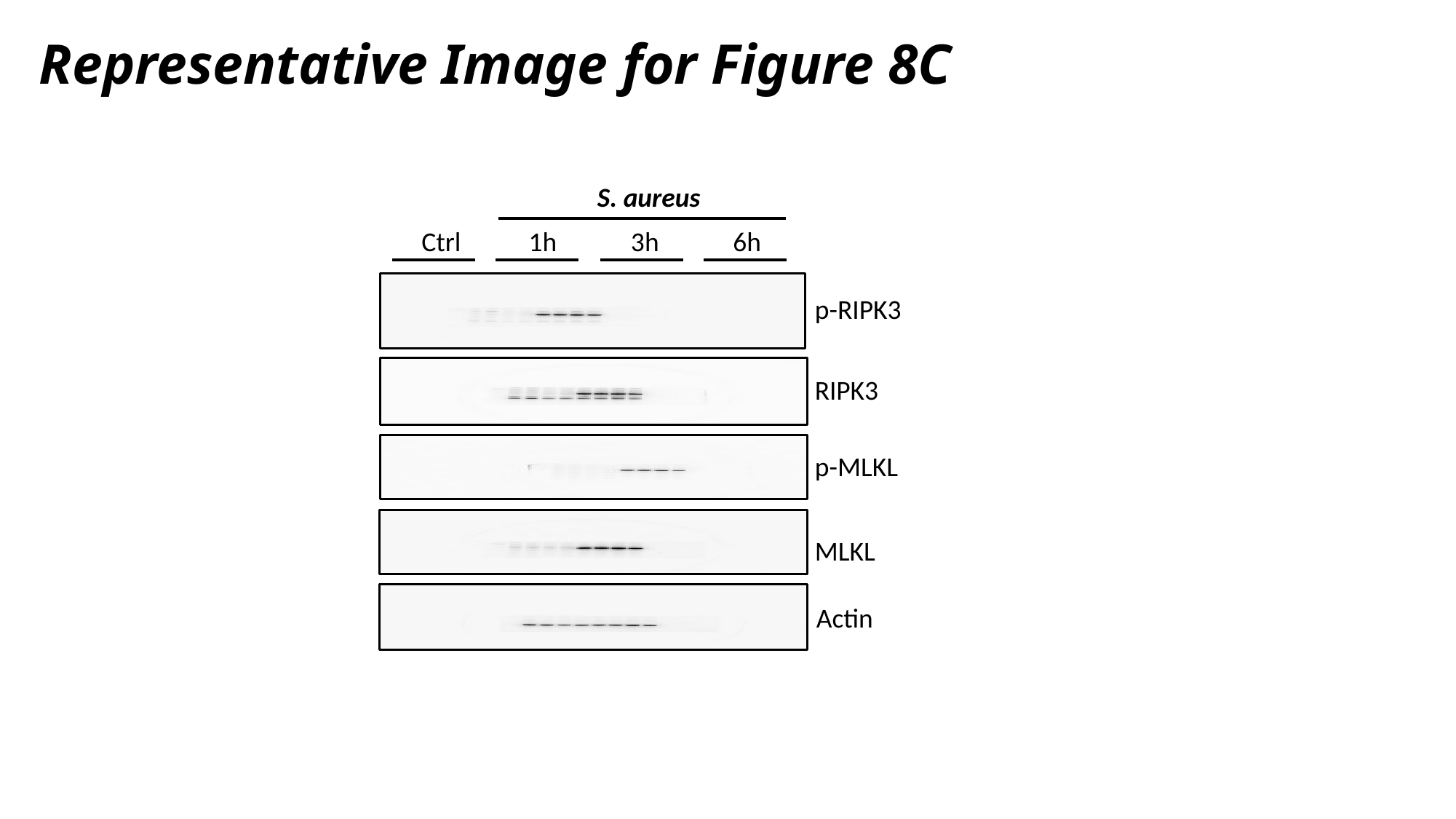

# Representative Image for Figure 8C
S. aureus
Ctrl 1h 3h 6h
p-RIPK3
RIPK3
p-MLKL
MLKL
Actin

## Slide 2
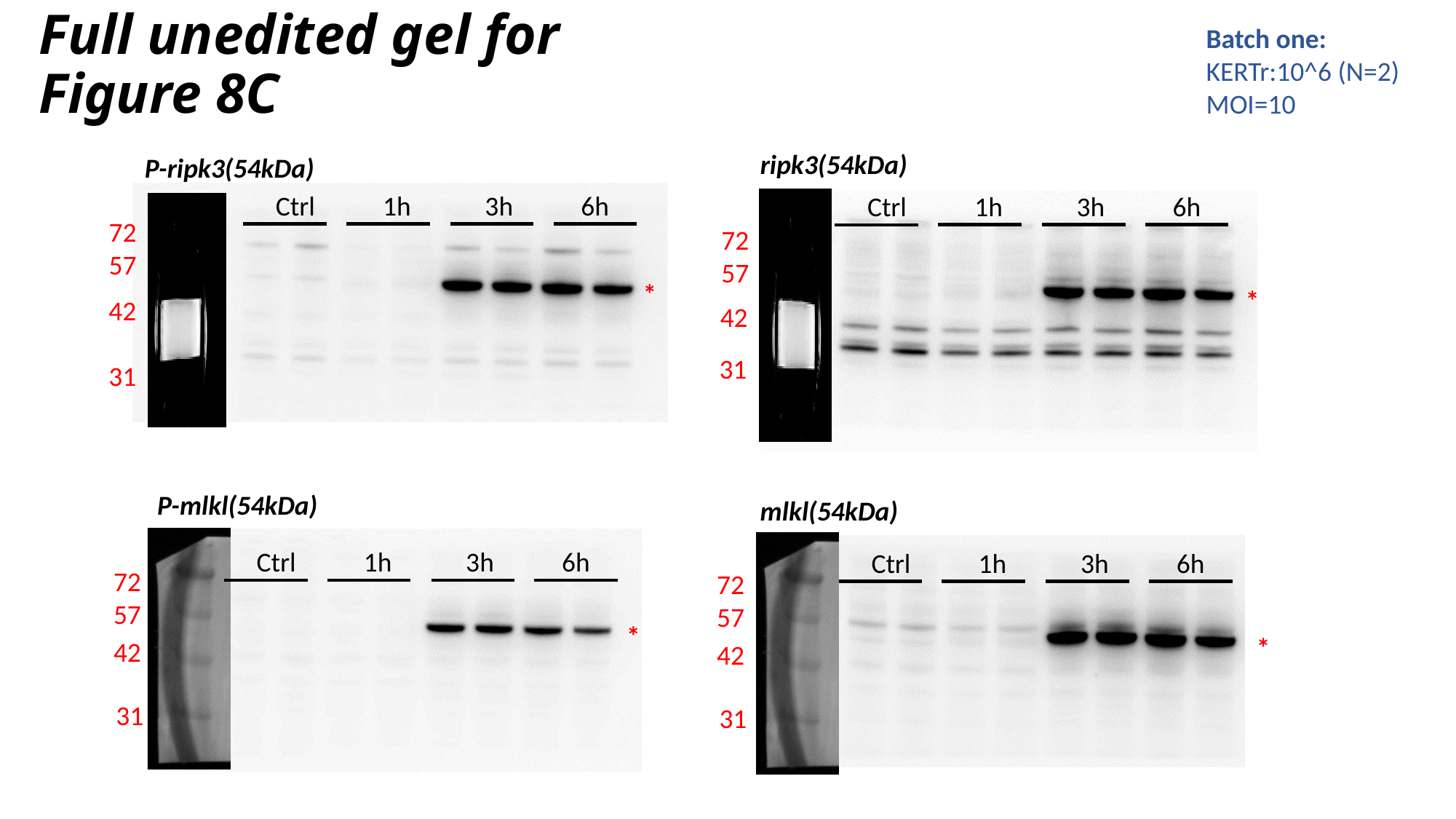

# Full unedited gel for Figure 8C
Batch one:
KERTr:10^6 (N=2)
MOI=10
ripk3(54kDa)
P-ripk3(54kDa)
Ctrl 1h 3h 6h
Ctrl 1h 3h 6h
72
57
72
57
*
*
42
31
42
31
P-mlkl(54kDa)
mlkl(54kDa)
Ctrl 1h 3h 6h
Ctrl 1h 3h 6h
72
57
72
57
*
*
42
42
31
31

## Slide 3
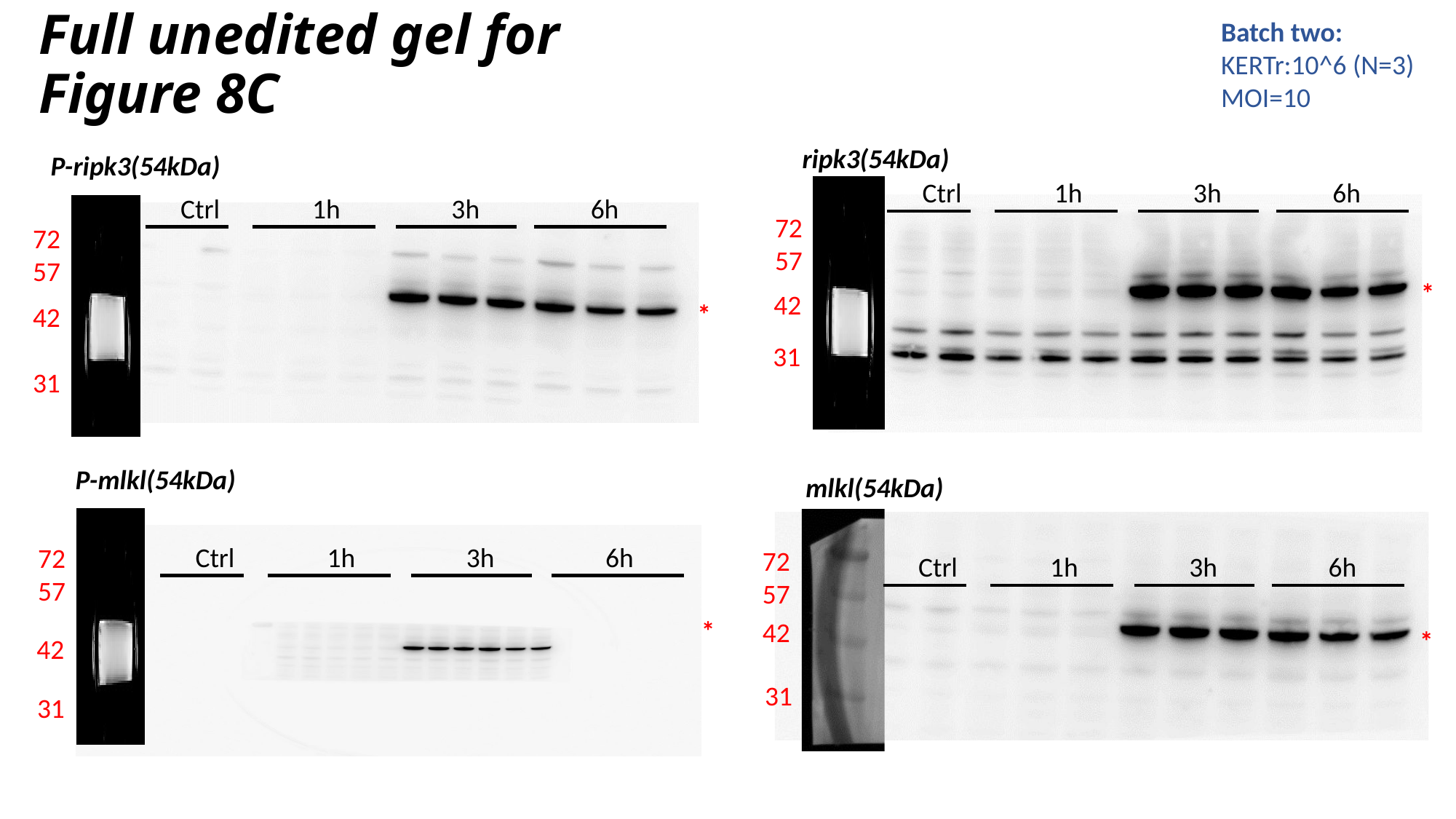

# Full unedited gel for Figure 8C
Batch two:
KERTr:10^6 (N=3)
MOI=10
ripk3(54kDa)
P-ripk3(54kDa)
Ctrl 1h 3h 6h
Ctrl 1h 3h 6h
72
57
72
57
*
42
*
42
31
31
P-mlkl(54kDa)
mlkl(54kDa)
Ctrl 1h 3h 6h
72
57
72
57
Ctrl 1h 3h 6h
*
42
*
42
31
31

## Slide 4
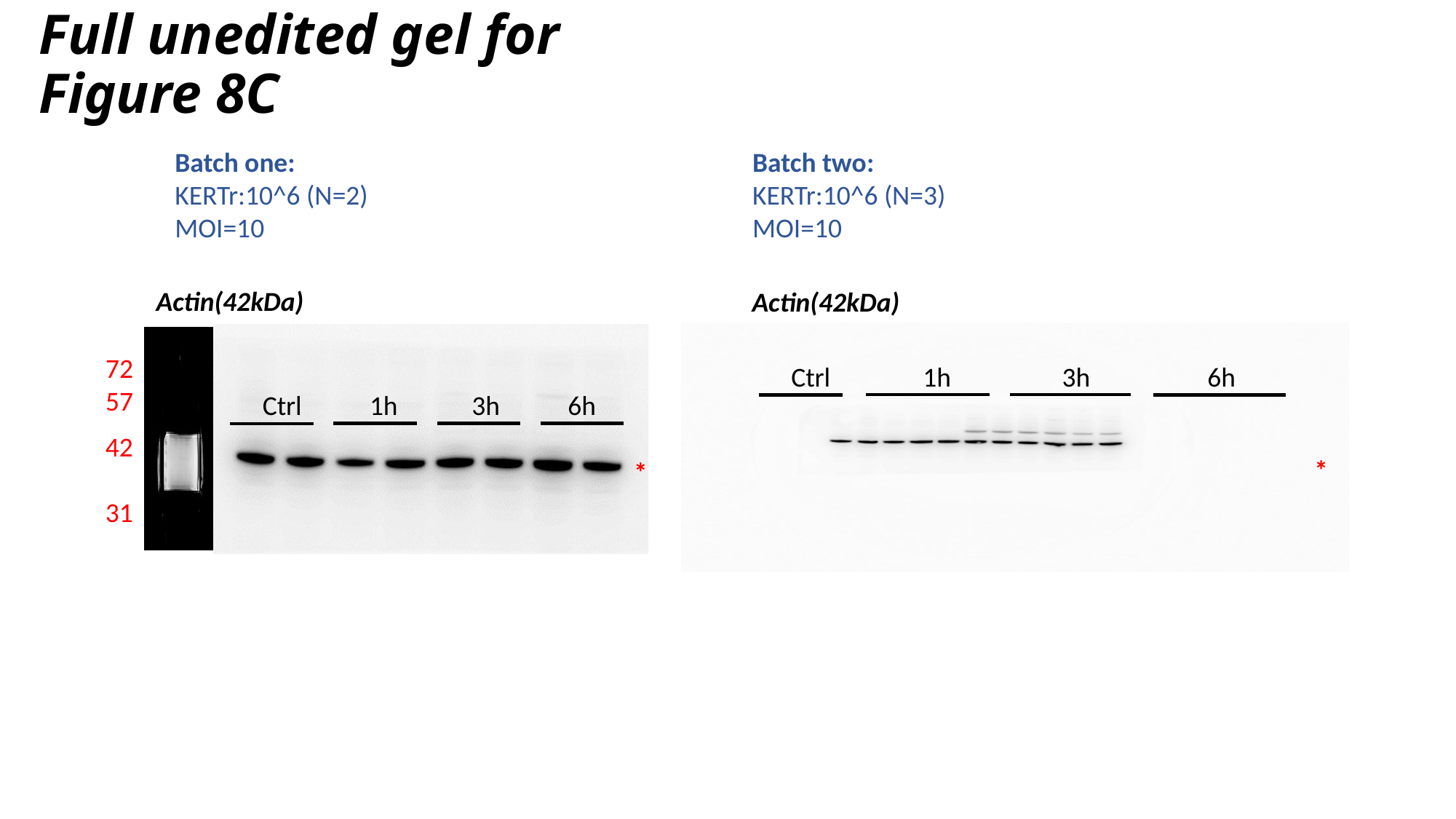

# Full unedited gel for Figure 8C
Batch one:
KERTr:10^6 (N=2)
MOI=10
Batch two:
KERTr:10^6 (N=3)
MOI=10
Actin(42kDa)
Actin(42kDa)
72
57
Ctrl 1h 3h 6h
Ctrl 1h 3h 6h
42
31
*
*
